# Preadipocyte IL-13/IL-13R**α**1 signaling regulates beige adipogenesis through modulation of PPAR**γ** activity

**DOI:** 10.1172/JCI169152

**Published:** 2025-04-08

**Authors:** Alexandra R. Yesian, Mayer M. Chalom, Nelson H. Knudsen, Alec L. Hyde, Jean Personnaz, Hyunjii Cho, Yae-Huei Liou, Kyle A. Starost, Chia-Wei Lee, Dong-Yan Tsai, Hsing-Wei Ho, Jr-Shiuan Lin, Jun Li, Frank B. Hu, Alexander S. Banks, Chih-Hao Lee

**Affiliations:** 1 Department of Molecular Metabolism, Harvard T.H. Chan School of Public Health, Boston, Massachusetts, USA.; 2 Graduate School of Biomedical Sciences, Department of Cellular, Molecular, and Developmental Biology, Tufts University School of Medicine, Boston, Massachusetts, USA.; 3 Genomics Research Center, Academia Sinica, Taipei, Taiwan.; 4 Graduate Institute of Immunology, National Taiwan University College of Medicine, Taipei, Taiwan.; 5 Department of Nutrition, Harvard T.H. Chan School of Public Health, Boston, Massachusetts, USA.; 6 Division of Endocrinology, Diabetes and Metabolism, Beth Israel Deaconess Medical Center, Harvard Medical School, Boston, Massachusetts, USA.

**Keywords:** Cell biology, Metabolism, Adipose tissue, Glucose metabolism, Obesity

## Abstract

Type 2 innate lymphoid cells (ILC2s) regulate the proliferation of preadipocytes that give rise to beige adipocytes. Whether and how ILC2 downstream Th2 cytokines control beige adipogenesis remain unclear. We used cell systems and genetic models to examine the mechanism through which IL-13, an ILC2-derived Th2 cytokine, controls beige adipocyte differentiation. IL-13 priming in preadipocytes drove beige adipogenesis by upregulating beige-promoting metabolic programs, including mitochondrial oxidative metabolism and PPARγ-related pathways. The latter was mediated by increased expression and activity of PPARγ through the IL-13 receptor 1 (IL-13R1) downstream effectors STAT6 and p38 MAPK, respectively. *Il13*-KO or preadipocyte *Il13ra1*-KO mice were refractory to cold- or β3-adrenergic agonist–induced beiging in inguinal white adipose tissue, whereas *Il4*-KO mice showed no defects in beige adipogenesis. *Il13*-KO and *Il13ra1*-KO mouse models exhibited increased body weight and fat mass and dysregulated glucose metabolism but had a mild cold-intolerant phenotype, likely due to their intact brown adipocyte recruitment. We also found that genetic variants of human *IL13RA1* were associated with BMI and type 2 diabetes. These results suggest that IL-13 signaling–regulated beige adipocyte function may play a predominant role in modulating metabolic homeostasis rather than in thermoregulation.

## Abstract 2

Preadipocyte IL-13-IL-13Ra1 signaling promotes beige adipogenesis through regulating PPAR gamma activity. IL-13-mediated beige adipocyte function contributes to body weight maintenance and metabolic homeostasis

## Graphical abstract

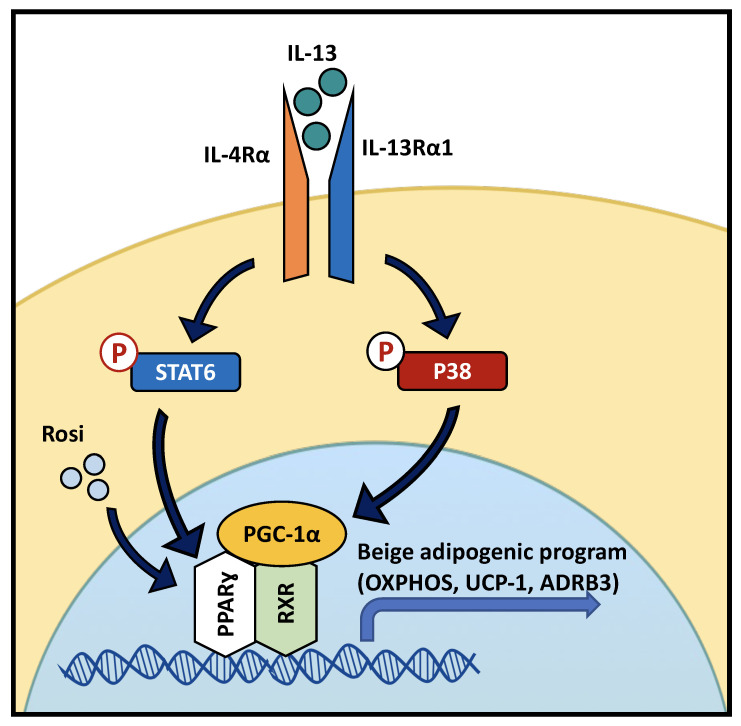

## Introduction

The maintenance of a healthy BMI throughout the lifespan is critical for prevention of the metabolic sequelae of obesity, including type 2 diabetes (T2D) (1). Relative to other fat depots, the subcutaneous white adipose tissue of humans has a high degree of metabolic flexibility that plays a key regulatory role in weight gain and glucose homeostasis (2). The inguinal white adipose tissue (iWAT) of mice similarly exhibits high metabolic activity, in part because it is a primary source of thermogenic beige adipocytes that undergo uncoupling protein 1–mediated (UCP1-mediated) uncoupled respiration upon sympathetic activation (2–4). In humans, the activity of thermogenic adipocytes is inversely associated with BMI and body fat percentage (5–7), and activation of these cells by β3-adrenergic agonists improves glucose tolerance in overweight patients (8). Studies have indicated that adult human thermogenic adipocytes may be more similar to murine beige adipocytes than murine brown adipocytes, making beige cells an appealing preclinical model for the development of new therapeutics (9, 10).

Inducible beige adipocytes initially develop via de novo adipogenesis, or recruitment (11–13). Although single-cell RNA-Seq studies have identified potential beige precursor populations (14–17), the delineation of factors that determine white versus beige adipocyte fate is ongoing. Several transcription factors and coactivators, such as PPARγ and PPARγ coactivator-1α (PGC-1α), have been implicated in both beige and brown adipogenic programs (18). The initiation of adipocyte differentiation requires activation of PPARγ, the master regulator of adipogenesis. In conventional preadipocyte cell models, such as 3T3-L1 cells that differentiate into large lipid droplet–filled mature adipocytes, *Pparg* expression can only be detected around the second day of a 6-day differentiation course (19). By contrast, *Pparg* has been identified as a marker of 1 beige precursor population, and activation of PPARγ by synthetic ligands has been shown to increase beiging of white adipose tissues (20–22). Most beige adipogenesis appears to occur in early postnatal development (23–25), and the proliferative capacity of adipogenic progenitor cells declines rapidly in mice between 4 and 7 weeks of age (25). After undergoing adipogenesis, mature beige adipocytes may “whiten” over time, as decreased stimulation reduces their thermogenic activity (2); however, these cells maintain their beige identity and have the potential to be reactivated in adulthood (13, 26, 27).

The Th2 cytokines IL-4 and IL-13, which share a heterodimeric receptor complex of IL-4Rα and IL-13Rα1 (28), are classically implicated in the immune response to allergens and parasitic worms (29). In mice, Th2 signaling has been proposed to control the development of beige adipose tissue (25, 30, 31). Notably, Th2 cytokines and type 2 innate lymphoid cells (ILC2s), which mainly produce IL-13 and IL-5, have been shown to regulate the proliferation of beige precursors in young mice and promote adaptive thermogenesis (25, 30). However, although one study suggests that the ILC2/Th2 cytokine axis regulates the proliferation of beige adipocyte progenitor cells and iWAT *Ucp1* expression in an IL-4Rα–dependent manner (25), a second study demonstrates that adipose ILC2s promote beiging through the production of methionine-enkephalin (30). Thus, questions remain as to whether the cytokines downstream of ILC2s are involved in beige adipogenesis and what mechanisms regulate this process.

We have previously shown that IL-13 acts directly on tissues and cells to regulate metabolism (32, 33). During endurance exercise, ILC2-derived IL-13 and muscle IL-13R1 promote the adaptive metabolic response by enhancing mitochondrial biogenesis and oxidative metabolism (32). In line with the metabolism-regulatory roles of IL-13 signaling, GWAS have identified *IL13RA1* as a top locus for BMI and T2D (34, 35). In this study, we demonstrate that, in addition to the reported function of Th2 cytokines in beige precursor proliferation (25), activation of preadipocyte IL-13/IL-13Rα1 signaling drove beige adipogenesis in iWAT, in part through regulation of PPARγ expression and activity. This regulatory mechanism did not affect brown adipocyte recruitment and, as such, was not required for maintaining core body temperature. Disruption of preadipocyte IL-13 signaling led to increased weight gain and insulin (Ins) resistance, suggesting that IL-13/IL-13Rα1–regulated beige adipogenesis may function to modulate metabolic homeostasis. Our results also demonstrate that IL-4 was not required for cold-induced beige adipocyte recruitment, highlighting the importance of the ILC2/IL-13 axis in white adipose tissue beiging during cold challenge.

## Results

### IL-13 signaling mediates beige adipocyte recruitment.

**Figure 1:**
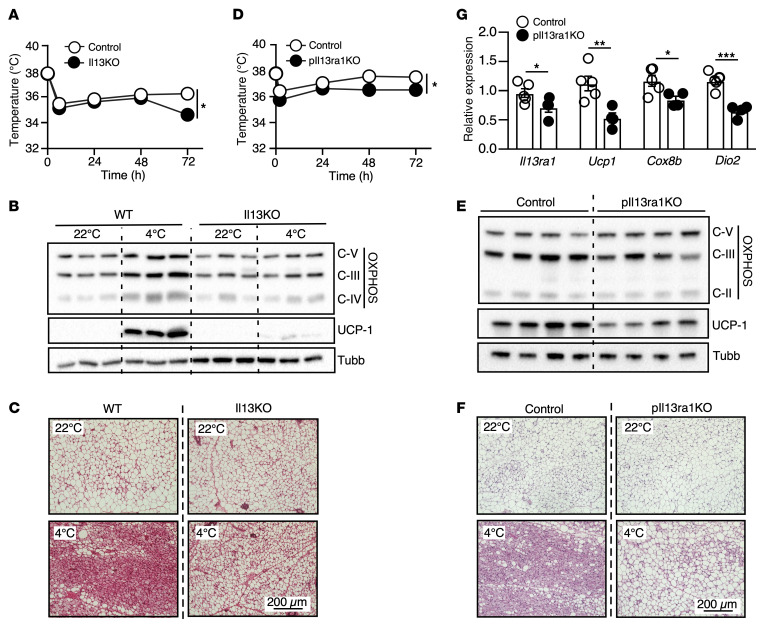
IL-13/IL-13R1 regulates beige adipocyte recruitment. (**A**) Core body temperature of 8-week-old female WT and *Il13*-KO mice during a 72-hour cold challenge at 4°C. *n* = 6 WT mice; *n* = 5 *Il13*-KO mice. The experiment was repeated in 2 separate cohorts. (**B**) Immunoblots showing protein levels of UCP1 and mitochondrial OXPHOS complexes III (UQCRC2), IV (MTCO1), and V (ATP5A) in iWAT of WT and *Il13*-KO mice. Representative samples from 3 mice/group are shown. (**C**) Representative H&E staining of iWAT from the mice in **A**. Scale bar: 200 μm. (**D**) Core body temperature of 5- to 7-week-old control and *pIl13ra1*-KO mice during the cold challenge at 4°C. *n* = 5/group. The experiment was performed in 1 cohort. (**E**) Immunoblots showing protein levels of UCP1 and mitochondrial OXPHOS complexes II, III, and V in iWAT of control and *pIl13ra1*-KO mice after the cold exposure in **D**. Representative samples from 4 mice/group are shown. (**F**) Representative H&E staining of iWAT and (**G**) mRNA expression of *pIl13ra1* and thermogenic genes measured by RT-qPCR in subcutaneous adipose tissue of the cold-exposed control and *pIl13ra1*-KO mice in **D**. *n* = 5/group. Scale bar: 200 μm. All values are presented as the mean ± SEM. *P < 0.05, **P < 0.01, and ***P < 0.001, by 2-way ANOVA (**A** and **D**) and 2-tailed, unpaired *t* test (**G**). Tubb, tubulin (loading control).

To examine the contributions of IL-13 signaling to thermogenesis in postnatal development, 8-week-old female WT and *Il13*-KO mice were subjected to a cold challenge. *Il13*-KO mice exhibited a significant reduction in the core body temperature after 72 hours at 4°C (Figure 1A). Immunoblot analysis of iWAT revealed a defect in the induction of UCP1 and mitochondrial oxidative phosphorylation (OXPHOS) complex proteins in cold-exposed *Il13*-KO mice compared with WT controls (Figure 1B). Histology further demonstrated a reduction in adipocyte beiging in iWAT of cold-exposed *Il13*-KO mice (Figure 1C). In contrast, there was no difference in tissue morphology or UCP1 protein expression in brown adipose tissue (BAT) of *Il13*-KO mice (Supplemental Figure 1, A and B; supplemental material available online with this article; https://doi.org/10.1172/JCI169152DS1), indicating that the thermogenic defect at the later stage of the cold challenge was likely due to impaired beige adipocyte development.

Since previous studies have reported that Th2 cytokines directly promote the proliferation of beige progenitor cells (25), we set out to determine whether IL-13 exerts its effects through preadipocyte IL-13Rα1 signaling. Mice with a conditional *Il13ra1* allele (*Il13ra1^fl/fl^*) were crossed with mice expressing a *Prx1^Cre^* transgene to generate preadipocyte *Il13ra1*-KO (*pIl13ra1*-KO) mice. *Prx1^Cre^* has previously been validated for the study of preadipocytes (36). PCR analyses of genomic DNA validated *Il13ra1* gene deletion in iWAT (and primary inguinal adipocytes) and, to a lesser extent, in epididymal white adipose tissue (eWAT) and BAT of *pIl13ra1*-KO mice (Supplemental Figure 1C). Young *Il13ra1^fl/fl^* (control) and *pIl13ra1*-KO male mice were subjected to a cold challenge. Similar to *Il13*-KO mice, *pIl13ra1*-KO mice exhibited a moderate defect in core body temperature maintenance and a reduction in iWAT UCP1 and OXPHOS proteins at 4°C (Figure 1, D and E). The morphology of iWAT from cold-exposed *pIl13ra1*-KO mice also showed impaired formation of multilocular beige adipocytes (Figure 1F). The BAT of *pIl13ra1*-KO mice appeared fully functional, as evidenced by UCP1 immunoblot and histologic analyses (Supplemental Figure 1, D and E). In concert, we observed decreased *Ucp1* and OXPHOS gene expression in iWAT, but not BAT, of *pIl13ra1*-KO mice, despite the reduction in *Il13ra1* expression detected in both fat depots (Figure 1G and Supplemental Figure 1F). *Il13ra1* expression in the liver and muscle was comparable between control and *pIl13ra1*-KO mice (Supplemental Figure 1G). Collectively, these results demonstrate that IL-13/IL-13Rα1 signaling in preadipocytes contributed to cold-induced recruitment of beige adipocytes.

IL-4 has also been implicated in beige adipogenesis (25). Splenocytes from *Il4*-KO mice failed to express *Il4* when stimulated with phorbol myristate acetate and ionomycin, while the expression of *Il13* was not affected (Supplemental Figure 2A). Unlike *Il13*-KO mice, *Il4*-KO mice exhibited no defects in body temperature maintenance after 72 hours at 4°C (Supplemental Figure 2B). In iWAT, the expression of *Ucp1* and OXPHOS genes and proteins and the formation of multilocular beige adipocytes were similar between WT and *Il4*-KO mice (Supplemental Figure 2, C–E). Similarly, histological and expression analyses demonstrated that cold-induced browning of BAT was not affected in *Il4*-KO mice (Supplemental Figure 2F and data not shown). To assess potential differences in temporal effects of IL-4 and IL-13 during cold-induced beige adipocyte recruitment, WT mice (7–8 weeks old) were given antibodies against IL-13 or IL-4 two hours before and 24 and 48 hours after initiation of a cold challenge. The short-term neutralizing antibody treatments did not affect cold tolerance over 72 hours (Supplemental Figure 2G), but anti–IL-13 antibody treatment significantly suppressed the upregulation of *Ucp1* and *Atp5k* by cold in iWAT compared with control IgG treatment (Supplemental Figure 2H). Immunoblots and histology revealed that iWAT UCP1 and beige adipocyte recruitment was also blunted by anti–IL-13 treatment (Supplemental Figure 2, I and J). These effects were not observed with anti–IL-4 treatment, except for the significant reduction of cold-induced iWAT UCP1 protein. Both anti–IL-13 and anti–IL-4 treatments had no effect on brown adipocyte recruitment (Supplemental Figure 2K and data not shown). These findings suggest that IL-4 was not required for beige adipogenesis and highlight the importance of IL-13 in cold-induced beiging of iWAT in mice.

### IL-13/IL-13Rα1 signaling in preadipocytes enhances the oxidative capacity of mature beige adipocytes.

To investigate whether IL-13/IL-13Rα1 signaling in preadipocytes promotes beige adipogenesis, immortalized clonal preadipocyte cell lines were generated from the iWAT of WT mice. Several clonal cell lines were characterized, all of which shared similar responses to differentiation and IL-13 treatment. For subsequent studies, we focused on the WT clone B6 preadipocytes (referred to as WT preadipocytes/cells), which differentiated robustly, with increasing expression of adipogenic and thermogenic markers over the course of 5–6 days (Supplemental Figure 3, A and B). The expression of *Il13ra1* declined after differentiation (Supplemental Figure 3C); similarly, we found that *Il13ra1* expression was lower in primary adipocytes compared with preadipocytes isolated from iWAT (Supplemental Figure 3D), indicating that IL-13Rα1 may have a predominant role in preadipocytes. We established stable shRNA-mediated *Il13ra1* knockdown (sh*Il13ra1*) or a control shRNA against luciferase (shLuc) in WT preadipocytes (Supplemental Figure 3E). Both stable lines differentiated comparably, as determined by Oil Red O staining, triglyceride (TG) content, and *Adipoq* gene expression, suggesting that *Il13ra1* was not required for adipocyte differentiation. (Supplemental Figure 3, F and H).

**Figure 2:**
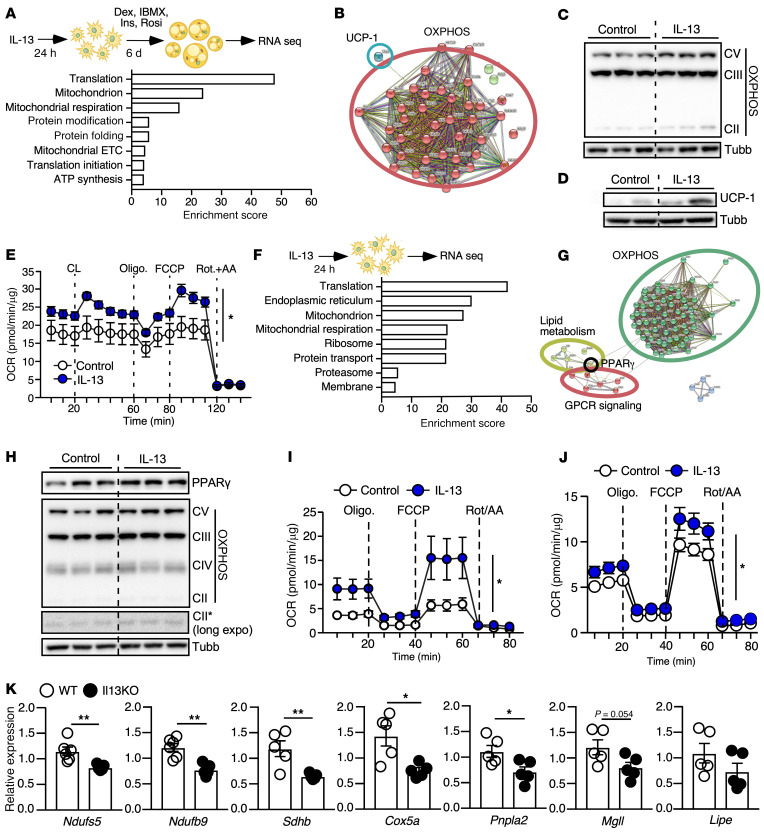
Regulation of mitochondria-related metabolic programs by IL-13 in preadipocytes enhances the oxidative capacity of mature adipocytes. (**A**) WT preadipocytes were treated with IL-13 or vehicle for 24 hours before induction of differentiation for 6 days, followed by RNA-Seq analysis. The top enriched categories upregulated by IL-13 pretreatment are shown. RNA-Seq was performed once but was repeated in a separate clonal line (*n* = 4). Bioinformatics was processed using the CLC Genomics Workbench. (**B**) STRING protein-protein interaction map of genes in the KEGG thermogenesis pathway upregulated by IL-13 pretreatment. (**C**) Immunoblotting of mitochondrial OXPHOS complex proteins in WT adipocytes (*n* = 3; 3-day differentiation; experiments were repeated twice) and (**D**) UCP1 protein in mature WT adipocytes (*n* = 2; 6-day differentiation) with or without IL-13 pretreatment. (**E**) Mitochondrial respiration of mature WT adipocytes with or without IL-13 pretreatment. CL, CL316,243. *n* = 10; experiments were repeated 3 times. (**F**) WT preadipocytes were treated with IL-13 or vehicle for 24 hours, followed by RNA-Seq. The top enriched categories upregulated by IL-13 are shown (*n* = 4). (**G**) STRING protein-protein interaction map of genes in the KEGG thermogenesis pathway upregulated by IL-13 treatment in preadipocytes. (**H**) Immunoblotting showing PPARγ and mitochondrial OXPHOS complex proteins by IL-13 in WT preadipocytes. *n* = 3/group. Experiments repeated more than 3 times. (**I**) Mitochondrial respiration of WT preadipocytes treated with IL-13 for 24 hours. *n* = 5; experiments were repeated 3 times. (**J**) Mitochondrial respiration of primary preadipocytes treated with IL-13 for 24 hours. *n* = 10; experiments were repeated twice. (**K**) RT-qPCR analyses to assess the expression of OXPHOS and PPARγ target genes in iWAT of WT and *Il13*-KO mice. *n* = 6 WT and 5 *Il13*-KO 8-week-old female mice. *P <0.05 and **P < 0.01, by 2-way ANOVA (**E**, **I**, and **J**) and 2-tailed, unpaired *t* test (**K**).

Preadipocytes were treated with IL-13 or vehicle for 24 hours before undergoing differentiation for 6 days. We performed RNA-Seq on differentiated adipocytes (Figure 2A). Using an FDR threshold of less than 0.01, we identified 318 differentially expressed genes in mature adipocytes that underwent IL-13 pretreatment compared with vehicle control (Supplemental Data File 1). To increase the power for gene enrichment analyses, the cutoff was reduced to a *P* value of less than 0.01, which yielded 629 genes upregulated by IL-13 pretreatment. Functional annotation clustering performed using the Database for Annotation, Visualization, and Integrated Discovery (DAVID) (37, 38) revealed 2 major categories of upregulated genes – one involved in translation and ribosomal function and another involved in mitochondrial function and respiration (Figure 2A and Supplemental Data File 1). Within the mitochondria category, we noted a subset of 42 genes in the Kyoto Encyclopedia of Genes and Genomes (KEGG) thermogenesis pathway (Supplemental Data File 1). A protein-protein interaction map generated using the STRING database demonstrated that the main cluster of the regulated genes included all 5 complexes of the mitochondrial electron transport chain (ETC) in the OXPHOS pathway and *Ucp1* (Figure 2B), suggesting that IL-13 treatment in the preadipocyte stage led to differentiation toward a beige adipocyte-like phenotype. In fact, IL-13 priming was sufficient to increase the levels of mitochondrial ETC complexes (Figure 2C) and upregulate UCP1 protein (Figure 2D) in differentiated adipocytes. The oxygen consumption rate (OCR) and phosphorylation of protein kinase A (PKA) substrates in response to stimulation with the β3-adrenergic agonist CL 316,243 (CL) were also higher in IL-13–primed adipocytes (Figure 2E and Supplemental Figure 3I). Regulation of mitochondrial function by IL-13 pretreatment was mediated by IL-13R1, as the induction of mitochondrial OXPHOS genes was lost in sh*Il13ra1* cells (Supplemental Figure 3J). RNA-Seq analysis also identified 797 downregulated genes, with protein modification and transcription comprising the major categories, including several homeobox genes known for their functions in development (Supplemental Data File 1).

To examine the mechanism by which IL-13 acts in preadipocytes to regulate beige adipogenesis, we analyzed RNA-Seq results in WT preadipocytes treated with IL-13 for 24 hours without differentiation. We identified 1,875 genes that were significantly upregulated and 1,797 genes that were significantly downregulated by IL-13 treatment in preadipocytes (FDR <0.01, Supplemental Data File 2). Functional annotation clustering yielded results similar to those obtained in the mature adipocyte analysis, with ribosomal and mitochondrial genes comprising the majority of upregulated categories (Figure 2F and Supplemental Data File 2). Protein modification and transcription comprised most downregulated categories (Supplemental Data File 2), including Notch signaling, which has been reported to suppress beige adipogenesis (39). The upregulated mitochondrial cluster included a subset of 81 genes in the KEGG thermogenesis pathway (Supplemental Data File 2). The protein-protein interaction map identified 3 major clusters, including clusters for lipid metabolism (e.g., *Pnpla2*, *Mgll*, and *Lipe*) and GPCR signaling molecules (e.g., *Gnas* and *Adcy1*) that are also utilized by the β3-adrenergic receptor (ADRB3), as well as for mitochondrial oxidative metabolism (e.g. *Ndufa1*, *Sdhb*, *Uqcrh*, *Cox6a1*, and *Atp5k*, Figure 2G). IL-13–treated preadipocytes exhibited an increase in OXPHOS proteins (Figure 2H), an effect sustained throughout differentiation (Supplemental Figure 3K). IL-13 treatment also enhanced the OCR in preadipocytes (Figure 2I), and this effect was maintained 2 days after differentiation (Supplemental Figure 3L). IL-13 similarly increased the OCR in primary cells derived from the stromal-vascular fraction (SVF) of WT iWAT (Figure 2J). In support of these cell-based findings, expression of mitochondrial OXPHOS and lipid metabolism genes was downregulated in the iWAT of 8-week-old *Il13*-KO mice compared with expression in WT control mice (Figure 2K). To further examine whether preadipocyte IL-13 signaling is important for cold-induced beige adipogenesis, control and whole-body *Il13ra1*-KO) mice were exposed to 4°C for 3 days; primary preadipocytes were isolated from iWAT, followed by adipocyte differentiation for 6 days. Cold exposure enhanced expression of *Il13ra1* and *Ucp1* in differentiated WT adipocytes compared with the room temperature (RT) condition (Supplemental Figure 3M). By contrast, the expression of *Ucp1* was lower in differentiated *Il13ra1*-KO adipocytes in both RT and cold-primed conditions compared with WT controls. These data indicate that preadipocyte IL-13 signaling drove differentiation into a highly oxidative beige adipocyte–like population.

### IL-13 acts upstream of PPARγ to regulate beige adipogenesis.

**Figure 3:**
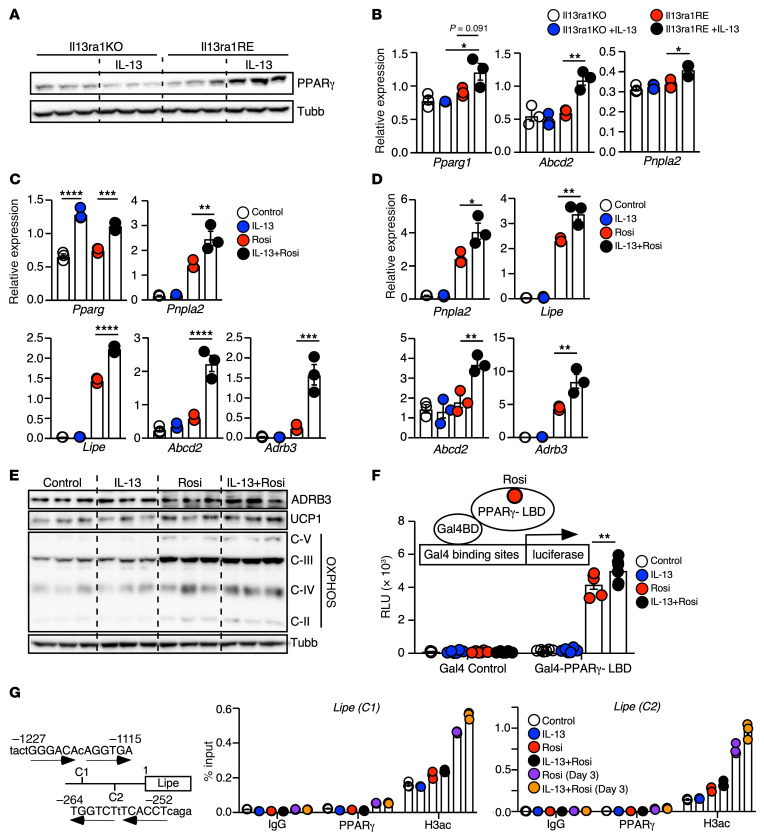
IL-13 potentiates PPARγ-mediated beige adipogenesis. (**A**) Immunoblot of PPARγ protein in *Il13ra1*-KO and *Il13ra1*-RE preadipocytes treated with IL-13 or vehicle for 24 hours. *n* = 3, experiment repeated twice. (**B**) RT-qPCR of *Pparg1* and PPARγ target genes in *Il13ra1*-KO and *Il13ra1*-RE preadipocytes treated with or without IL-13 for 24 hours. *n* = 3, experiment performed 3 times. (**C**) RT-qPCR of Pparg and PPARγ target genes in WT preadipocytes with indicated treatments for 24 hours. *n* = 3, experiment performed 4 times. (**D**) RT-qPCR analyses of WT cells with indicated treatments for 24 hours, followed by 2 days of differentiation. *n* = 3, experiments performed 3 times. (**E**) Immunoblotting in WT cells with indicated treatments for 24 hours, followed by differentiation for 5 days. *n* = 3/condition, experiments repeated twice. (**F**) WT preadipocytes were transfected with a Gal4 control or Gal4-PPARγ-LBD expression vector, together with a Gal4-binding site containing luciferase reporter and a β-gal internal control. Graph shows PPARγ LBD transactivation on the luciferase reporter with indicated treatments for 24 hours. Luciferase activity was normalized to β-gal activity to determine the RLU. *n* = 4, experiments performed 3 times. (**G**) Schematic shows 2 sets of ChIP primer pairs (C1 and C2) flanking 2 potential PPREs on the Lipe gene promoter ( the transcriptional start site designated as 1). Arrows indicate 2 direct repeats of the PPRE. Graphs show ChIP analyses of preadipocytes treated overnight with indicated treatments and of adipocytes differentiated for 3 days after overnight Rosi or IL-13+Rosi pretreatment using antibodies against the IgG control, PPARγ, or H3ac. *n* = 3 technical replicates, experiments performed 3 times. *P < 0.05, **P < 0.01, ***P < 0.001, and ****P < 0.0001, by 2-way ANOVA with Tukey’s multiple-comparison test (**B**) and 1-way ANOVA with Tukey’s multiple-comparison test (**C** and **D**).

The 3 clusters of IL-13–regulated pathways in the protein-protein interaction map converged on PPARγ as a regulatory node (Figure 2G). In fact, the lipid metabolism cluster included several PPARγ target genes, such as hormone-sensitive lipase (HSL; *Lipe*)*,* adipose triglyceride lipase (ATGL; *Pnpla2*)*,* monoglyceride lipase (*Mgll*)*,* seipin (*Bscl2*)*,* and ATP-binding cassette subfamily D member 2 (*Abcd2*). We first validated that IL-13 treatment increased PPARγ protein in the WT preadipocyte cell line (Figure 2H) and in primary cells derived from the SVF of WT iWAT (Supplemental Figure 4A). To determine potential clonal effects from the WT preadipocyte cell line and assess the role of IL-13R1 in the control of *Pparg* expression, we also generated preadipocyte clonal cell lines from the iWAT of *Il13ra1*-KO mice. Multiple *Il13ra1*-KO preadipocyte clones were characterized, and the KO clone 1.5 cell line was utilized to generate a transgenic *Il13ra1*-reexpressing (*Il13ra1*-RE) cell line for rescue studies (Supplemental Figure 4B). *Il13ra1*-RE cells differentiated comparably to control cells with pBabe empty vector (*Il13ra1*-KO control, Supplemental Figure 4, C and D). Although IL-13 was ineffective in *Il13ra1*-KO cells, the regulation of *Pparg* and its downstream targets was restored in the *Il13ra1*-RE preadipocytes, demonstrating that induction of the PPARγ pathway by IL-13 was IL-13R1 dependent (Figure 3, A and B). Of note, preadipocytes only expressed *Pparg1*; expression of *Pparg2* was detectable at the later stage of adipocyte differentiation and was lower than that of *Pparg1* (data not shown). We obtained the same reverse transcription quantitative PCR (RT-qPCR) results using primer pairs for *Pparg1* or *Pparg* (common region for *Pparg1* and *Pparg2*).

Activation of PPARγ by high-affinity ligands such as rosiglitazone (Rosi) promotes beige adipogenesis both in vivo and in cultured adipocytes (20, 21, 40–43). To explore the IL-13/PPARγ axis in beige adipogenesis, we treated preadipocytes with vehicle, IL-13, Rosi, or a combination of IL-13 plus Rosi (IL-13+Rosi) for 24 hours. Rosi treatment had no effect on *Pparg* gene expression but induced the expression of PPARγ target genes to a much greater degree than did IL-13 treatment (Figure 3C), which was expected from a potent PPARγ agonist. However, IL-13+Rosi treatment resulted in significantly higher expression of PPARγ target genes compared with Rosi alone. This effect persisted after switching to differentiation medium (containing Dexamethasone [Dex], Ins, and 3-isobutyl-10-methylxanthine [IBMX])for 2 days (Figure 3D). We observed a similar result for the expression of *Adrb3*, consistent with the increased CL-triggered OCR and signaling observed in mature adipocytes primed with IL-13 (Figure 2E and Supplemental Figure 3I). Furthermore, mature adipocytes that received IL-13+Rosi priming expressed higher levels of UCP1 and ADRB3 proteins than did those treated with Rosi alone (Figure 3E). Thus, Rosi priming in preadipocytes was sufficient to promote beige adipogenesis, and IL-13 potentiated PPARγ-mediated differentiation of beige adipocytes with enhanced β3-adrenergic signaling.

The effect of Rosi+IL-13 on PPARγ target gene expression could be explained by the approximately 2-fold increase in *Pparg* expression with IL-13 treatment (Figure 3C and Supplemental Figure 4A). However, the regulation of some genes, notably *Abcd2*, appeared to be synergistic. This prompted us to investigate whether IL-13/IL-13Rα1 signaling further modulates PPARγ activity. To examine this potential mechanism, we induced overexpression of *Pparg1* in WT preadipocytes (Pparg1-OE). In the pBabe empty vector control cells, IL-13 treatment significantly induced *Pparg* gene expression (Supplemental Figure 4E). Pparg1-OE preadipocytes had higher *Pparg* gene expression under unstimulated conditions, which did not further increase with IL-13 treatment. However, Pparg1-OE cells treated with IL-13+Rosi still expressed higher levels of PPARγ target genes compared with those treated with Rosi alone, suggesting that IL-13 signaling may also enhance PPARγ activity in preadipocytes (Supplemental Figure 4F). To quantitatively assess PPARγ transcriptional activity, we transfected Pparg1-OE preadipocytes with a PPARγ response element (PPRE) luciferase reporter construct. The cells were treated with vehicle control, IL-13, Rosi, or IL-13+Rosi for 24 hours. PPRE activity was significantly higher in IL-13+Rosi–treated cells than in cells treated with Rosi alone (Supplemental Figure 4G). Using a mammalian 1-hybrid system, in which the PPARγ ligand–binding domain (LBD) was fused to yeast Gal4 DNA–binding domain (Gal4-BD–PPARγ–LBD, Figure 3F), we demonstrated that IL-13 similarly enhanced Rosi-induced PPARγ transactivation of the luciferase reporter activity in WT preadipocytes. Of note, IL-13 alone did not affect PPARγ activity, indicating that IL-13 treatment did not lead to the production of endogenous PPARγ ligands. In line with a nonessential role for IL-4 in beige adipogenesis, IL-4 treatment in preadipocytes failed to increase mitochondrial respiration and to potentiate the effects of Rosi on the regulation of *Lipe* expression and Gal4-BD–PPARγ–LBD transactivation activity (Supplemental Figure 4, H–J).

We performed ChIP on the promoters of 2 PPARγ target genes, *Lipe* and *Mgll*, to examine PPARγ DNA–binding activity. We identified 2 potential PPREs (designated as C1 and C2) within 1.3 kb of the promoter region of the *Lipe* gene (Figure 3G). The upstream C1 site, but not the downstream C2 site, showed similar PPARγ binding at day 3 of differentiation in Rosi- or IL-13+Rosi–primed cells (Figure 3G). However, Rosi priming increased the signal of the acetyl-histone H3 Lys27 (H3ac) on both C1 and C2 sites after the overnight treatment, and to a greater extent on day 3 of differentiation, which was further enhanced by IL-13+Rosi cotreatment. For the *Mgll* gene, 2 adjacent PPREs were identified at around 2.5 kb of the 5′ promoter region (Supplemental Figure 4K). PPARγ binding was also detected on both sites on day 3 of differentiation in Rosi- or IL-13+Rosi–primed cells (Supplemental Figure 4K). IL-13+Rosi also induced a stronger effect than did Rosi alone on increasing H3ac signal in the ChIP assay. Collectively, these results suggest that IL-13 did not affect PPARγ ligand production or DNA binding, but instead may have modulated PPARγ transcriptional activity, likely through coactivator-mediated regulation of the chromatin landscape.

### IL-13/IL-13R1 potentiates PPARγ transcriptional activity through p38 MAPK.

**Figure 4:**
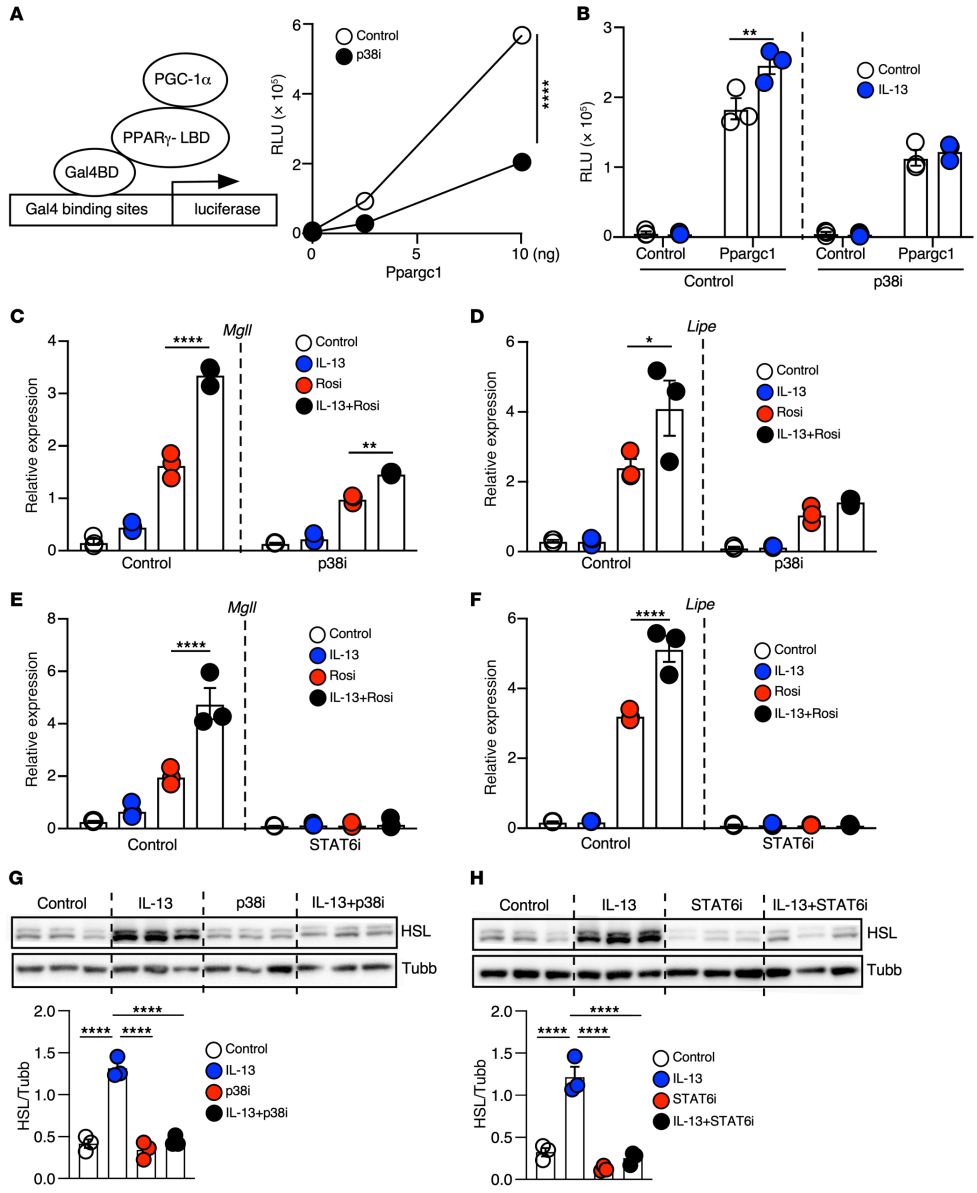
IL-13/IL-13R1 increases the expression and activity of PPARγ through STAT6 and p38 MAPK. (**A**) Schematic of the 1-hybrid system to assess PGC-1α coactivation on PPARγ LBD activity. AD293 cells were transfected with Gal4-PPARγ-LBD and *Ppargc1* expression vector, together with Gal4 binding site containing a luciferase reporter and β-gal as an internal control. Graph shows quantification of PPARγ LBD transactivation on the luciferase reporter in AD293 cells cotransfected with 0, 2.5, or 10 ng *Ppargc1a* expression vector in the presence of vehicle or P38i (10 μM). Luciferase activity was measured 48 hours after transfection and normalized to β-gal activity to determine the RLU. *n* = 3. The experiment was performed 3 times. (**B**) Quantification of PPARγ LBD transactivation in AD293 cells cotransfected with luciferase/β-gal reporters, Gal4-PPARγ-LBD, and a control vector or *Ppargc1a* expression vector (10 ng). Cells were treated with vehicle, IL-13, P38i, or IL-13+P38i overnight. RLU was determined 48 hours after transfection. *n* = 3. The the experiment was performed 3 times. (**C** and **D**) Expression of PPARγ target genes measured by RT-qPCR in WT preadipocytes treated with IL-13, Rosi, or IL-13+Rosi for 24 hours with or without P38i. *n* = 3. The experiment was performed twice. (**E** and **F**) Expression of PPARγ target genes by RT-qPCR in WT preadipocytes treated with IL-13, Rosi, or IL-13+Rosi for 24 hours ± STAT6i (10 μM). *n* = 3. The experiment was performed once. (**G**) Immunoblotting showing HSL protein in preadipocytes treated with IL-13+Rosi or vehicle with or without P38i for 24 hours. *n* = 3, with quantifications shown. The experiment was performed twice. (**H**) Immunoblotting showing HSL protein in WT preadipocytes treated with IL-13+Rosi or vehicle with or without STAT6i for 24 hours. *n* = 3, with quantifications shown. The experiment was performed twice. *P < 0.05, **P < 0.01, ***P < 0.001, and ****P < 0.0001, by 2-way ANOVA (**A** and **B**), 2-way ANOVA with Tukey’s multiple-comparison test (**C**–**F**), and 1-way ANOVA with Tukey’s multiple-comparison test (**G** and **H**).

PGC-1α, a well-established PPARγ coactivator and beige adipocyte regulator, can be activated by p38 MAPK in response to inflammatory cytokines (44). IL-13 has been shown to activate p38 MAPK in immune cells (45). In preadipocytes, IL-13 induced the phosphorylation of p38 MAPK in a time course comparable to that of the TLR4 ligand LPS (Supplemental Figure 5A). To assess whether IL-13 could increase PPARγ activity through the p38/MAPK/PGC-1 axis, we continued our studies in AD293 cells because of their low background PPARγ activity. In AD293 cells, *Ppargc1* cotransfection dose dependently enhanced the activity of PPARγ LBD even in the absence of Rosi, and this effect was blunted by SB 203580, a p38 MAPK inhibitor (p38i) (Figure 4A). In addition, IL-13 significantly increased PGC-1–mediated PPARγ LBD activity, an effect abolished by p38i (Figure 4B). Similar results were obtained in WT preadipocytes (Supplemental Figure 5B). In the presence of p38i, IL-13 still significantly induced *Pparg* gene expression (Supplemental Figure 5C). However, gene expression analyses in WT cells demonstrated that p38i blocked the potentiation activity of IL-13+Rosi cotreatment on PPARγ target genes (Figure 4, C and D). This inhibitory effect of p38i treatment in preadipocytes persisted 3 days after differentiation (Supplemental Figure 5D).

STAT6, the canonical downstream regulator of Th2 cytokines, increases *Pparg* expression in macrophages (46, 47). In preadipocytes, IL-13 robustly induced phosphorylation of STAT6 (Supplemental Figure 5A). Treatment with AS1517499, a STAT6 inhibitor (STAT6i), blocked the induction of *Pparg* expression by IL-13 (Supplemental Figure 5E). STAT6i also significantly reduced the expression of PPARγ target genes (Figure 4, E and F), an effect maintained after 3 days of differentiation (Supplemental Figure 5F). STAT3 has also been shown to regulate oxidative metabolism downstream of IL-13 in muscle (32). In preadipocytes, IL-13 was able to increase STAT3 phosphorylation, which appeared to decline quickly compared with phosphorylated STAT6 (p-STAT6) (Supplemental Figure 5G). Treatment with a STAT3i (Stattic) failed to inhibit the IL-13–mediated increase in PPARγ target gene expression (Supplemental Figure 5H), indicating that STAT3 was not a primary mediator of IL-13 signaling in preadipocytes.

Consistent with the gene expression result, Rosi+IL-13 cotreatment induced HSL protein expression to a greater extent than did Rosi in WT preadipocytes (Figure 4G). p38i cotreatment reduced the induction of HSL protein by IL-13. Furthermore, differentiated adipocytes primed with IL-13+Rosi expressed higher levels of UCP1 and UQCRC2 (OXPHOS complex III) proteins compared with Rosi alone (Supplemental Figure 5I), and the effect of IL-13 was blunted by p38i cotreatment. STAT6i similarly suppressed HSL protein expression (Figure 4H). In addition, STAT6i treatment in preadipocytes completely blocked differentiation (data not shown), likely due to PPARγ inhibition. Collectively, these data indicate that intact PPARγ signaling in preadipocytes played an important role in beige adipogenesis and that IL-13 promoted beige adipocyte differentiation by modulating both the expression and activity of PPARγ through the STAT6 and p38 pathways, respectively.

### IL-13/IL-13Rα1 signaling in mature adipocytes is not required for thermogenesis.

**Figure 5:**
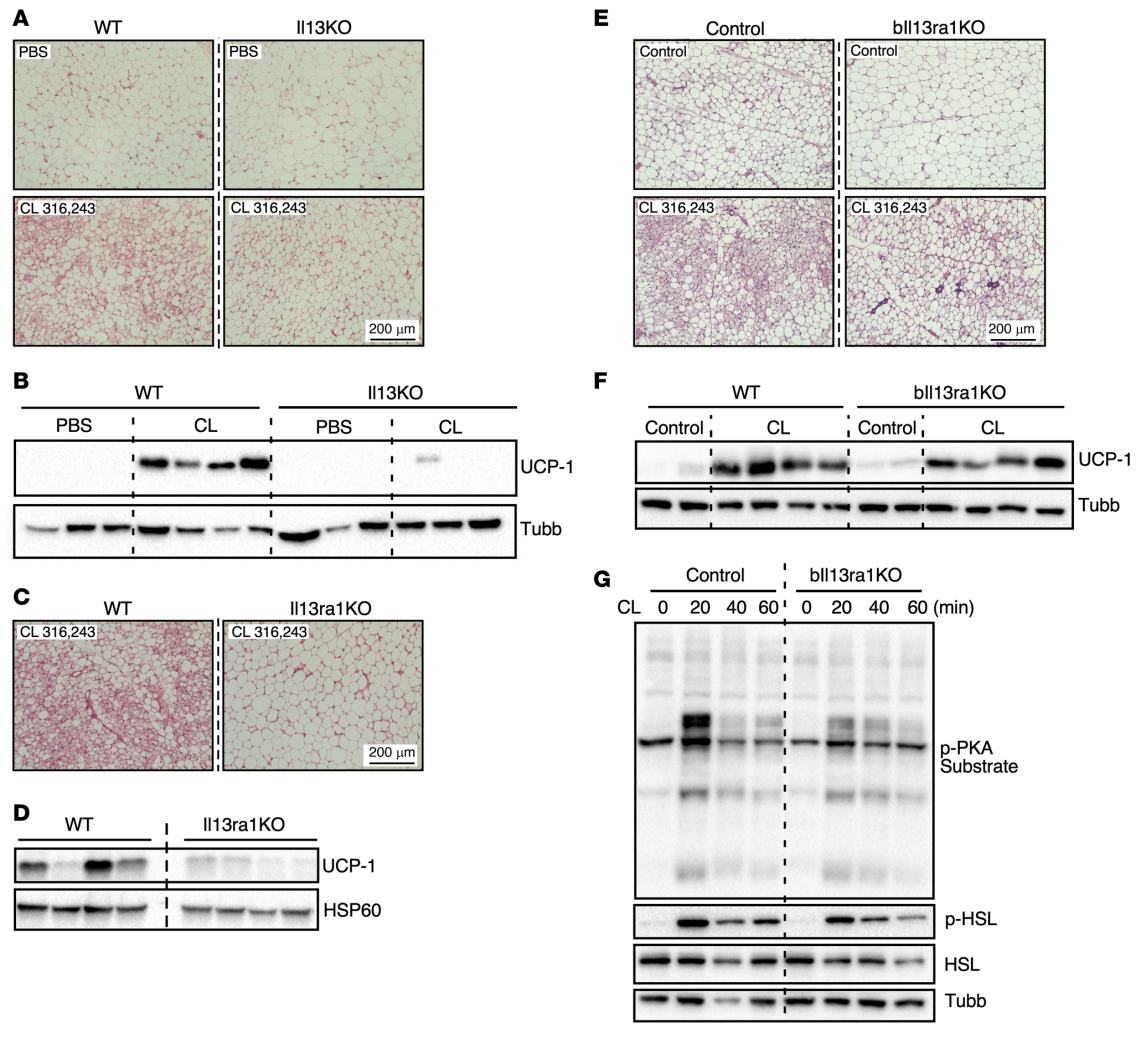
Adult mice deficient in IL-13 signaling exhibit impaired responses to β3-adrenergic stimulation. (**A**) Representative H&E staining of iWAT from WT and *Il13*-KO mice injected with PBS or CL for 10 days. *n* = 3–4/group (3-month-old males). The experiment was performed once. (**B**) Immunoblotting showing UCP1 protein levels in iWAT of the WT and *Il13*-KO mice in **A**. Tubulin was used as a loading control. (**C**) H&E staining and (**D**) immunoblotting of iWAT from control and *Il13ra1*-KO mice injected with CL for 10 days. *n* = 4 (30-week-old males). For H&E-stained images, samples from 1 mouse of each group are shown, with HSP60 used as a loading control. (**E**) H&E staining and (**F**) immunoblotting of iWAT from control and *bIl13ra1*-KO mice injected with or without CL once daily for 7 consecutive days. *n* = 3 for noninjected control mice; *n* = 6–7 for CL-injected mice (5-month-old females). The experiment was performed once. Representative tissue samples are shown. (**G**) Immunoblotting for HSL, p-HSL (S660), and p-PKA substrates in iWAT explants from control and *bIl13ra1*-KO mice. Tissue was stimulated with CL for 0, 20, 40, and 60 minutes ex vivo. Pooled analysis of 2 mice/genotype (5-month-old females). The experiment was performed 3 times. Scale bars: 200 μm (**A**, **C**, and **E**).

Previous studies have demonstrated that beige precursor cells decline in adult mice (25). Thus, we investigated the effects of IL-13 deficiency on the thermogenic competence of adult mice, and found no difference in the body temperature between WT and *Il13*-KO mice at thermoneutrality, 22°C, or 4°C (Supplemental Figure 6A). However, *Il13*-KO mice ate significantly more at 4°C during the light cycle. Similar results were observed in *Il13ra1*-KO mice (Supplemental Figure 6B). The increased food intake may indicate a compensatory effect to maintain body temperature and suggests that IL-13/IL-13R1 may still modulate beige adipocyte function in adult mice. To test this possibility, we pharmacologically activated the beige/brown adipogenic program by dosing WT and *Il13*-KO mice with CL for 10 days. Histology demonstrated that *Il13*-KO mice had larger adipocytes in iWAT under unstimulated conditions and showed impaired formation of multilocular adipocytes in response to CL (Figure 5A). Immunoblots also revealed a diminished induction of UCP1 in response to CL in the iWAT of *Il13*-KO animals (Figure 5B). Consistent with findings in young mice, histological and immunoblot analyses of BAT revealed no difference in tissue morphology or UCP1 expression between CL-injected WT and *Il13*-KO mice (Supplemental Figure 6, C and D). *Il13ra1*-KO mice exhibited a similar defect in CL-induced beige adipocyte recruitment and UCP1 protein expression (Figure 5, C and D).

The attenuated response to CL in adult *Il13*-KO and *Il13ra1*-KO mice could be due to 2 nonmutually exclusive scenarios: decreased beige adipogenesis in postnatal development, resulting in a smaller population of mature cells to be activated in adulthood, or decreased beige adipocyte activation due to loss of IL-13 signaling in mature adipocytes. To clarify the role of IL-13 in promoting beige cell activation, we generated mature beige/brown adipocyte *Il13ra1*-KO mice by crossing *Il13ra1^fl/fl^* mice with *Ucp1^Cre^* mice. We detected the deleted *Il13ra1* genomic DNA in iWAT and BAT, and the deletion was further enhanced by cold exposure, in which *Ucp1* was expected to be highly induced (Supplemental Figure 6E). A reduction in *Il13ra1* mRNA expression was detected in mature adipocytes, but not the SVF from iWAT, indicating the *bIl13ra1*-KO model left preadipocyte IL-13 signaling intact (Supplemental Figure 6F). In line with this notion, we found that 6-week-old *bIl13ra1*-KO mice had no defect in thermogenesis after 72 hours at 4°C compared with control *Il13ra1^fl/fl^* mice (Supplemental Figure 6G). There was also no difference in tissue morphology of iWAT and BAT between control and *bIl13ra1*-KO mice upon cold exposure (Supplemental Figure 6, H and I).

In 5-month-old control mice, a 7-day CL treatment induced the formation of multilocular beige adipocytes and increased UCP1 protein levels in iWAT (Figure 5, E and F). These responses appeared to be reduced in *bIl13ra1*-KO mice, though to a lesser extent than in *Il13*-KO, *Il13ra1*-KO, and *pIl13ra1*-KO mice. To further examine the CL/β3-adrenergic response, iWAT fat pads from control and *bIl13ra*-KO mice were stimulated with CL ex vivo. Immunoblotting confirmed that the induction of both p-PKA substrates and p-HSL by CL was reduced in *bIl13ra1*-KO fat pads compared with control mouse fat pads (Figure 5G). Collectively, these results suggest that, while IL-13/IL-13R1signaling may serve a functional role in UCP1^+^ mature adipocytes from iWAT, IL-13 exerted its beige adipocyte–promoting activity primarily in preadipocytes.

### The Il13ra1 gene is associated with body weight and glucose metabolism.

**Figure 6:**
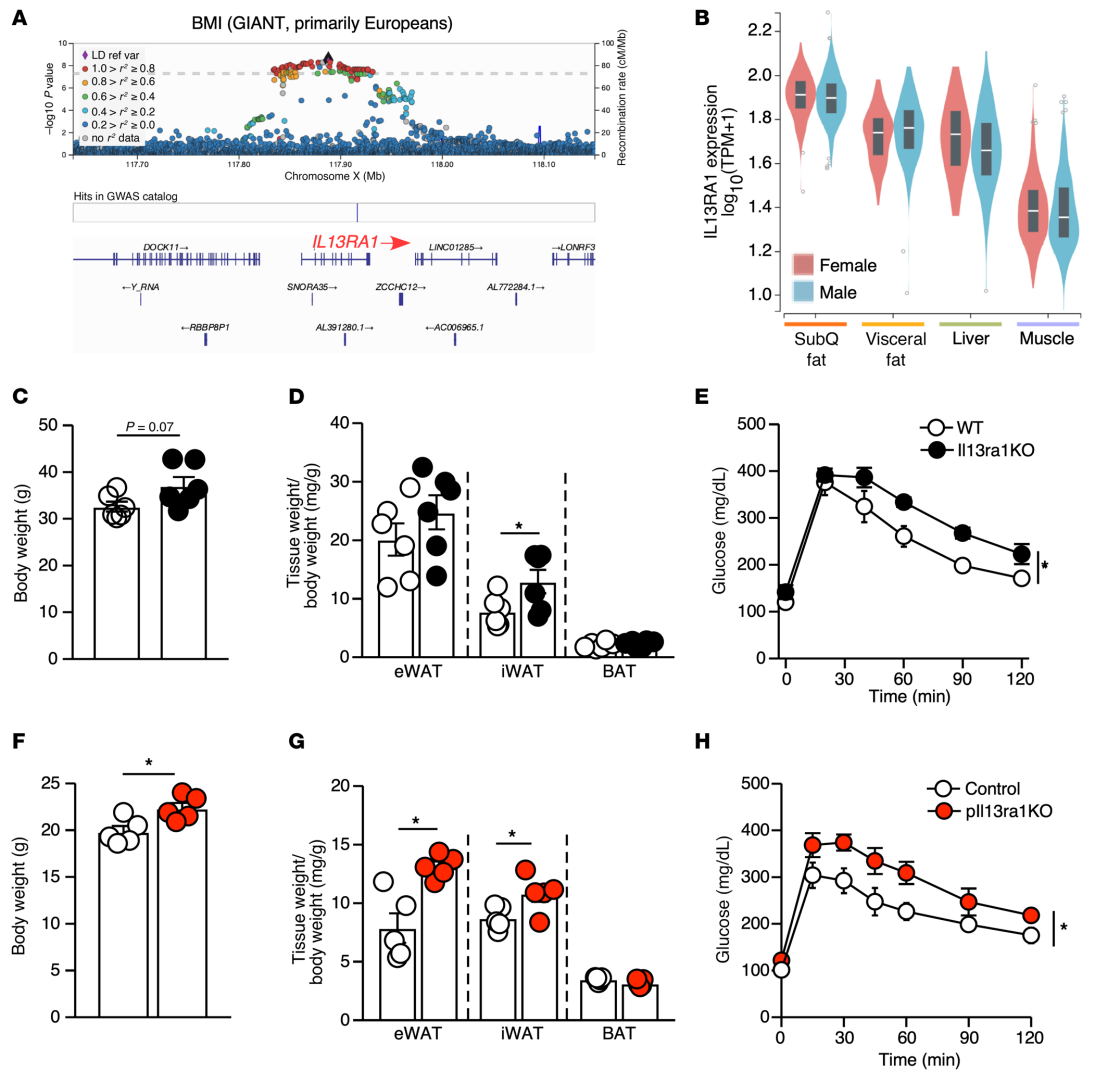
*Il13ra1* is associated with body weight. (**A**) Variants located at the *IL13RA1* gene showing genome-wide significant associations with BMI in a multiethnic population, based on X chromosome GWAS results from a GIANT Consortium study. The regional association plot was generated by LocusZoom (https://my.locuszoom.org/) (66) on –log_10_ *P* values for variant-trait associations. Each dot is a variant; the diamond-shaped dot indicates the lead variant with the smallest *P* value. Dot colors indicate LD relationships (*r^2^*) between all variants and the lead variants. The minor allele frequency is not included. (**B**) Gene expression levels of *IL13RA1* (log-transformed transcripts per million) in 4 human tissues, in women and men, based on data from the GTEx Portal (50). (**C**) Body weight and (**D**) fat tissue weight normalized to the body weight of WT and *Il13ra1*-KO mice. *n* = 6/genotype (20-week-old males). (**E**) Intraperitoneal glucose tolerance test (GTT) of WT and *Il13ra1*-KO mice. *n* = 5–6 per group (5-month-old mice). Experiments in **C**–**E** were repeated in 2 separate cohorts. (**F**) Body weight and (**G**) fat tissue weight normalized to the body weight of control and *pIl13ra1*-KO mice. *n* = 5/group (5- to 7-week-old males). (**H**) GTT of WT and *pIl13ra1*-KO mice. *n* = 7/group (20-week-old females). The experiment was performed in 1 cohort. *P < 0.05, by 2-tailed, unpaired *t* test (**C**, **D**, **F**, and **G**) and 2-way ANOVA (**E** and **H**).

In a large-scale GWAS from Biobank Japan (34, 35), variants of *IL13RA1* were significantly associated with BMI and T2D (Supplemental Figure 7, A and B). Genetic variants at the *IL13RA1* locus were also associated with BMI in the Genetic Investigation of Anthropomorphic Traits (GIANT) Consortium (a multiethnic group composed of 62.2% European, 16.4% Hispanic/Latin American, 15.3% Asian, and 6.1% African American/Afro-Caribbean individuals, Figure 6A) (48); however, the same locus was not associated with T2D in the predominately European population (data not shown) (49). Variants located on *IL13RA2* (a decoy receptor for IL-13) or *IL4R* were not associated with BMI or T2D (Supplemental Figure 7, C and D, and data not shown). Expression levels of human *IL13RA1* were higher in subcutaneous fat compared with levels in visceral fat, liver, and muscle (50) (Figure 6B).

In mice, loss of *Il13ra1* resulted in increased body weight and significantly heavier iWAT fat pads compared with WT mice (Figures 6, C and D). *Il13ra1*-KO mice were also more glucose intolerant (Figure 6E). A similar phenotype was observed in *pIl13ra1*-KO mice compared with control mice (Figure 6, F–H). Given the relatively minor role of IL-13/IL-13R1 signaling in mature adipocytes, the fat mass and glucose tolerance findings in *bIl13ra1*-KO mice were unsurprisingly less profound compared with those for *pIl13ra1*-KO or *Il13ra1*-KO mice (Supplemental Figure 7, E–G). These data suggest that IL-13/IL-13Rα–mediated beige adipocyte function may play a regulatory role in weight maintenance and metabolic homeostasis.

## Discussion

Using multiple mouse models and cell systems, we demonstrate that preadipocyte IL-13/IL-13Rα1 signaling was required for beige adipogenesis. IL-13 priming in preadipocytes was sufficient to drive the development of highly oxidative beige adipocytes, and the effects of IL-13 pretreatment were maintained throughout differentiation. PPARγ acted as one of the major downstream effectors of IL-13/IL-13Rα1 signaling; IL-13 increased the expression of PPARγ through STAT6 and enhanced PPARγ activity by p38 MAPK-mediated PGC-1α coactivation. Despite its role in regulating beige adipogenesis, deletion of IL-13 signaling, either through *Il13* or *Il13ra1* gene deletion, yielded a moderate defect in thermogenesis. This is consistent with another report suggesting that the thermogenic capacity of beige adipocytes in iWAT represents approximately 10% of that of brown adipocytes in C57BL/6 mice (51). Our published work on *Il13*-KO (33) and the current study demonstrate that mice lacking whole-body or preadipocyte IL-13/IL13Rα1 signaling show increased weight gain and glucose intolerance. These findings thus uncouple the thermogenic and metabolic functions of IL-13–regulated beige adipogenesis.

Th2 cytokines have been reported to promote beige cell development by enhancing the proliferation of PDGFRα^+^ adipocyte progenitors (25). Given the limited quantity of progenitors, characterizing the mechanistic role of Th2 signaling in beige adipogenesis has been challenging. Through the development of immortalized cell lines from iWAT, our study identifies an additional role for IL-13 in promoting the commitment of preadipocytes to beige adipocyte differentiation. IL-13 priming enhances oxygen consumption and OXPHOS complex protein expression in both preadipocytes and mature adipocytes. We have previously demonstrated that IL-13 controls muscle mitochondrial biogenesis and oxidative metabolism in response to endurance exercise training (32), suggesting that mitochondrial function is a primary target of IL-13 signaling. While STAT3 appears to mediate the effect of IL-13 in muscle, IL-13 regulates beige adipogenesis partly through STAT6 and PPARγ. Previous studies have identified *Pparg* as a marker for beige preadipocytes (22). In conventional preadipocyte cell models, such as 3T3-L1 cells, *Pparg* expression is induced 2 days after initiation of differentiation (19). The iWAT-derived preadipocyte cell lines established in the current study expressed *Pparg* at an appreciable level in the undifferentiated state, and these cells differentiated robustly to become mature adipocytes with small lipid droplets and elevated mitochondrial OXPHOS protein levels (Supplemental Figure 3). In addition, treatment with Rosi or IL-13+Rosi for 24 hours in preadipocytes was sufficient to promote differentiation into *Ucp1*-expressing adipocytes (Figure 3E). These observations suggest that preadipocyte PPARγ expression and activity play an important role in beige adipogenesis.

PPARγ agonists, such as Rosi, have been used to induce beiging in cell and mouse studies. Since PPARγ is required for differentiation of all types of adipocytes, how does PPARγ activation exert the observed beiging effect? It is possible that modulation of PPARγ activity by transcriptional cofactors also contributes to beige adipogenic capacity (18). For instance, PGC-1, originally identified as a PPARγ coactivator, has been shown to drive thermogenic gene expression and promote mitochondrial biogenesis in multiple tissues (52). In adipose tissue, deletion of PGC-1α results in a decrease in thermogenic and oxidative gene expression in iWAT, but not BAT (53). We found that in preadipocytes, IL-13 not only upregulated PPARγ gene and protein expression but also increased its activity. The latter only required the PPARγ LBD, where ligand-induced coactivator recruitment occurs. In the presence of PGC-1α, IL-13 further increased PPARγ LBD activity in a reporter assay. We identified p38 MAPK as a downstream effector of IL-13/IL-13Rα1 signaling mediating PCG-1α activation. p38 MAPK activation by cold and β3-adrenergic stimulation has previously been identified as a regulator of *Ucp1* expression in BAT, and this effect was dependent on PGC-1α (54). Indeed, pharmacological inhibition of p38 blocked the ability of IL-13 to enhance PGC-1 coactivation of the PPARγ LBD. Similarly, p38 inhibition abrogated the synergistic effect of IL-13 and Rosi cotreatment on PPARγ target gene expression in preadipocytes. Furthermore, “translation” is one of the top pathways upregulated by IL-13 treatment, which contains many ribosomal genes (Supplemental Data File 2). In concert, the induction of HSL protein by IL-13 in the presence of Rosi (Figure 4G) was higher than the increase in *Lipe* mRNA (which encodes HSL) (Figure 4D), suggesting that IL-13 could regulate PPARγ targets through posttranscriptional regulation.

While previous work has demonstrated that both IL-4 and IL-13 promote the proliferation of beige precursors (25), *Il4*-KO mice showed no defect in cold-induced beige adipocyte recruitment in iWAT, suggesting that IL-4 was sufficient but not required for beige adipogenesis. It is also possible that within the first 3 days at 4°C, ILC2s and ILC2-derived IL-13 were the primary regulators of beige adipocyte differentiation, whereas IL-4– and IL-4–producing cells, such as eosinophils, may have become important after prolonged cold exposure. Our results indicate that the relevance of IL-13–regulated beige adipocyte recruitment in thermogenesis in young mice became evident only after prolonged cold exposure. Previous studies have indicated that Th2 cytokines are dispensable for thermogenesis in adult mice (25). Consistent with these reports, we found that 20-week-old mice lacking the IL-13/IL-13R1 pathway had no thermogenic defect, although these mice still had a blunted induction of beiging in response to β3-adrenergic stimulation. IL-13 signaling did not affect brown adipocyte function. It is possible that the intact BAT activity was sufficient to maintain core body temperature in *Il13*-KO, *Il13ra1*-KO, and *pIl13ra1*-KO mice. Interestingly, these mice gained more weight and had impaired glucose intolerance compared with control mice. These results seem to disassociate the thermogenic and metabolic effects of IL-13 signaling–mediated beige adipogenesis. The metabolic phenotypes were less evident in *bIl13ra1*-KO mice, which had deletion of the *Il13ra1* gene in *Ucp1*-expressing mature adipocytes, suggesting that IL-13 acted primarily in preadipocytes to regulate metabolism. Consistent with the observations in our mouse genetic models, genetic variants of the human *IL13RA1* gene have been shown to be associated with T2D and BMI in a Japanese population and with BMI in a predominately European population.

The human *PPARG* gene is among the first identified to associate with T2D and BMI (55, 56). PPARγ agonists are powerful antidiabetic medications; however, existing drugs that target PPARγ have undesirable side effects, including weight gain and bone resorption (57, 58). A recent mouse study demonstrated that genetic overexpression of preadipocyte PPARγ in the early postnatal period is protective against the development of glucose intolerance later in life, providing evidence that the regulation of preadipocyte PPARγ is worthy of mechanistic investigation (59). Although our data support the notion that PGC-1α coactivation is important for mediating the beige adipogenic process, it is possible that other transcriptional regulators and/or posttranslational modifications of PPARγ may also be involved in the effects downstream of IL-13/IL-13Rα1. Future studies aiming to improve the understanding of additional levels of regulatory mechanisms by the preadipocyte IL-13/PPARγ axis may help the development of more targeted therapeutics.

## Methods

### Sex as a biological variable.

Our study examined male and female animals, and similar findings are reported for both sexes.

### Animal studies.

Mice were housed in a barrier facility under a 12-hour light/12-hour dark cycle and ad libitum access to chow (Mouse Diet 20 5058, PicoLab) and water. *Il13*-KO mice on the BALB/c background were backcrossed with C57BL/6J mice (JAX, The Jackson Laboratory) as previously described (33). Generation of the *Il13ra1*-KO mouse line was previously described (32). To obtain conditional KO mice, the artificial exon acceptor sequences of the *Il13ra1^–/–^* allele were removed by crossing with the flipase transgene; these mice were used to generate cell type–specific KOs. *Il4*-KO mice (knockin huCD2 [KN2]) on the C57BL/6J background were generated by replacing the first 2 exons of *Il4* with a human CD2-encoding sequence (60) provided in-house. *Prx1-Cre* mice (JAX, strain 005584, The Jackson Laboratory) and *Ucp1-Cre* mice (JAX, strain 024670, The Jackson Laboratory) were used to generate *pIl13ra1*-KO and *bIl13ra1*-KO mice, respectively. The *Prx1-Cre* strain (61) and the *Ucp1-Cre* strain (62) have been characterized previously. A complete list of animal cohorts is found in Supplemental Table 1.

### Cold exposure.

Mice were transferred from 22°C and single-housed at 4°C for 72 hours under 12-hour light/12-hour dark cycles and ad libitum access to chow and water. Body temperatures were assessed using a rectal probe thermometer at baseline (22°C), and following 2, 4, 6, 24, 48, and 72 hours at 4°C. For neutralizing antibody experiments (63), mice were i.p. injected with anti–IL-13, anti–IL-4, or isotype IgG control at 100 g/animal 2 hours before cold exposure. Two additional doses at 50 g were given 24 and 48 hours after cold exposure. Tissue was harvested at 72 hours.

### Adipocyte cell line development and culture.

Primary SVF was harvested from inguinal fat pads of WT and *Il13ra1*-KO mice as described below. Preadipocytes were immortalized by retroviral infection with SV40 Large T antigen and maintained in high-glucose (4.5 g/L) DMEM (Corning) containing 10% FB Essence (Avantor) and penicillin-streptomycin (Corning). Multiple clonal cell lines were characterized in both the WT and *Il13ra1*-KO backgrounds. To generate stable knockdown cell lines, shRNA for *Il13ra1* was subcloned into the pSIREN-RetroQ vector and transfected into Phoenix packaging cells to produce a retrovirus. Selection was done with 3 μg/mL puromycin. The same protocol was used to generate stable OE cell lines for *Il13ra1* and *Pparg1* in the pBabe vector.

Preadipocytes were plated at a density of 5 × 10^4^ cells per well (12-well plates) and left overnight, followed by treatment with 10 ng/mL recombinant mouse IL-13 (or IL-4, R&D Systems), 1 μM Rosi (MilliporeSigma), or a combination of IL-13 (10 ng/mL) plus Rosi (1 μM) for 24 hours. The p38-MAPK inhibitor SB203580 (Abcam), the STAT6i AS1517499 (Selleck Chemicals), and the STAT3i Stattic (Abcam) were suspended in DMSO and administered at a concentration of 10 μM and 1 μM, respectively.

Two methods were used to differentiate preadipocytes. Preadipocytes were differentiated in high-glucose DMEM, 10% FBS (Gemini Bio-Products) with a complete cocktail of 5 μg/mL Ins from bovine pancreas (MilliporeSigma), 1 μM Rosi, 0.5 mM IBMX (MilliporeSigma), and 1 μM Dex for 2 days, followed by maintenance media containing 5 μg/mL Ins and 1 μM Rosi for 4 days; or, following priming with 1 μM Rosi for 24 hours, cells were differentiated in a cocktail of 5 μg/mL Ins, 0.5 mM IBMX, and 1 μM Dex for 2 days, followed by maintenance media containing 5 μg/mL Ins for 4 days.

### RNA-Seq and analysis.

RNA-Seq studies were conducted in 2 different immortalized clonal cell lines with similar results. RNA-Seq was performed on 4 replicates per condition (preadipocyte plus vehicle, preadipocyte plus IL-13, mature adipocyte plus vehicle pretreatment, or mature adipocyte plus IL-13 pretreatment). Sequencing, data processing, and preliminary analyses were conducted at the IMB Genomics Core and IMB Bioinformatics Service Core at the Academia Sinica (Taipei, Taiwan) as described previously (32, 64). Briefly, samples were quantified with RiboGreen (Life Technologies, Thermo Fisher Scientific), and RNA integrity was checked with a Bioanalyzer 2100 (Agilent Technologies) (RNA integrity number [RIN] >8; OD 260/280 and OD 260/230 >1.8). RNA libraries were prepared with the TruSeq Stranded mRNA Library Preparation Kit (Illumina). Sequencing was analyzed with an Illumina NextSeq 500. Raw data were analyzed using the CLC Genomics Workbench. A *P*-value cutoff of 0.01 and an FDR *P* value of 0.01 were used for analyses of mature adipocytes and preadipocyte studies, respectively. Gene ontology analysis was performed using DAVID (37, 38). Protein-protein interaction maps were generated using the STRING database (65).

### Primary preadipocyte isolation and culture.

Inguinal fat pads with lymph nodes excised were minced and transferred to a digestion buffer containing high-glucose DMEM with 2% BSA and 2 mg/mL collagenase II (Gibco, Thermo Fisher Scientific). Following tissue digestion with mechanical disruption for 1 hour at 37°C, the digestion media were filtered through a 70 μm or 100 μm mesh to remove debris. The cell suspension was pelleted by centrifugation. Mature adipocytes on the top layer were collected for separate assays. The remaining SVF was resuspended in ACK buffer to lyse RBCs, washed with DMEM, and resuspended in cell culture media containing high-glucose DMEM with 10% FB Essence and penicillin-streptomycin. Cells were expanded in 10 cm cell culture plates for 3 days before being transferred to 12-well plates for experiments.

### Mitochondrial respiration.

Measurement of the OCR was performed using a Seahorse XF24 Bioanalyzer. Preadipocytes were plated in XF24 microplates at a density of 2 × 10^4^ cells per well to attach overnight. Cells were treated with mouse recombinant IL-13 (or IL-4) at a dose of 10 ng/mL or with vehicle control for 24 hours. An hour before the assay, cells were washed twice with PBS and changed to media containing minimal DMEM, 5 mM glucose, and 1 mM sodium pyruvate. Basal respiration was measured 3 times. Cells were then treated with oligomycin (2 μM), trifluoromethoxy carbonylcyanide phenylhydrazone (FCCP) (1 μM), and rotenone (Rot) plus antimycin A (AA) (1 μM). Measurements were recorded 3 times following each injection. The OCR was normalized to total protein content. The same protocol was carried out on day 3 of adipocyte differentiation.

For day-5 OCR analyses, preadipocytes were plated in a 6-well plate and treated with Rosi (1 μM) or a combination of IL-13 (10 ng/mL) and Rosi (1 μM) for 24 hours. Cells were washed and differentiated for 2 days in complete media containing a cocktail of Ins, IBMX, and Dex. Cells were then replated in XF24 microplates at a density of 1.5 × 10^4^ per well. Differentiation was continued in complete media containing Ins for day 2 through day 5. One hour before the assay, cells were treated as described above. CL was injected at a final concentration of 2 μM, followed by treatments with oligomycin (4 μM), FCCP (2 μM), and Rot plus AA (1 μM). OCR was normalized to total protein content for each well.

### Gene expression.

RNA was isolated from cells using the NucleoSpin RNA Plus kit (Macherey-Nagel), and from tissue using TRIzol-like reagent. Reverse transcription of isolated RNA was performed using the Verso cDNA synthesis kit (Thermo Fisher Scientific). RT-qPCR using SYBR green (SMOBio) was performed to quantify gene expression. Gene expression was normalized to a standard curve and presented as expression relative to *36b4*. The primer sequences are listed in Supplemental Table 2.

### Immunoblotting.

Protein quantification of cell and tissue lysates was performed using a bicinchoninic acid (BCA) kit (Pierce, Thermo Fisher Scientific). Proteins were separated by SDS-PAGE, transferred onto PVDF membranes, and incubated with primary antibody overnight at 4°C in TBS with Tween 20 (TBST) with 1% BSA. The primary antibodies used are listed in Supplemental Table 3. The Bio-Rad ChemiDoc XRS+ imaging system was used to detect ECL signal. Quantification of immunoblot results was performed using ImageJ software (NIH).

### Reporter assays.

For PPRE (from the *Acox1* gene promoter) reporter assays, *Pparg1*-OE preadipocytes cultured in 10 cm plates were transfected with the PPRE reporter construct at a concentration of 10 g/10^6^ cells for 24 hours, replated on 24-well plates (~4 × 10^4^ cells/well), and treated overnight with IL-13, Rosi, or IL-13+Rosi. Cells were lysed using a passive lysis buffer (Promega), and the Promega Luciferase Assay System was used to quantify luciferase activity, which was normalized to protein content to obtain relative luciferase units (RLU). To assess PPARγ LBD activity, WT preadipocytes were cotransfected in 10 cm plates with the Gal4-binding site–containing reporter (10 g) and either Gal4-BD control or Gal4-PPARγ-LBD (2.5 μg). Twenty-four hours after transfection, each plate was split into two 10 cm plates and transfected overnight with either CMV vector control or *Ppargc1* expression vector (2.5 μg) before plating in 24-well or 48-well plates. After attachment, cells were treated overnight with IL-13, Rosi, or Rosi+IL-13 with or without the P38 inhibitor. In certain experiments, IL-4 treatment was included. The reporter luciferase activity was normalized to protein content for RLU. For AD293 cells, cotransfection was conducted directly in 96-well plates. A β-gal reporter was included for normalization to obtain RLU (50 ng for reporters and up to 10 ng for each expression vector).

### ChIP.

Assessments of PPARγ promoter occupancy and histone H3 lysine 27 acetylation (H3K27ac) were performed using the SimpleChIP plus kit (Cell Signaling Technology) according to the manufacturer’s instructions. Chromatin preparations from eight-to-ten 15 cm dishes of preadipocytes or adipocytes (differentiated for 3 days) were pulled for each treatment/condition. Five grams of chromatin was used for each ChIP reaction that included either the negative control normal rabbit IgG (Cell Signaling Technology, 2729), PPARγ (81B8) rabbit mAb (Cell Signaling Technology, 2443), or Lys27 (D5E4) XP rabbit mAb (Cell Signaling Technology, 8173). Eluted DNA samples were quantified using real-time PCR with primer pairs flanking 2 potential PPARγ-binding sites on the 5′ regulatory region of the *Lipe* or *Mgll* gene.

### Indirect calorimetry.

Monitoring of food intake, energy expenditure, and body temperature as done in a Comprehensive Laboratory Animal Monitoring System (CLAMS, Columbus Instruments). Temperature probes were implanted s.c. 1 week prior to the experiment. Mice were individually housed, and experiments were recorded continuously under an ambient housing temperature for 3 days at thermoneutrality (30°C), 3 days at RT (22°C), and 3 days in cold (4°C).

### CL injection and signaling.

Mice were administered CL (Tocris) in PBS at a dose of 1 mg/kg body weight via i.p. injection. Injections were performed once daily for 7–10 days. Mice were weighed every 2 days, and the dose of CL was adjusted accordingly. For ex vivo signaling, inguinal fat pads were isolated from 20-week-old mice and sliced bidirectionally using a brain slicer. The minced tissue was pooled by genotype, divided into 4 wells, and suspended in high-glucose DMEM. The tissue was acclimated for 10 minutes at 37°C with gentle shaking. CL (1 μM) was added for 0, 20, 40, and 60 minutes, after which tissue samples were washed with PBS and snap-frozen before processing for immunoblot analyses.

### TG assay.

Immortalized preadipocytes were plated and differentiated in 24-well plates. On day 6 of differentiation, cells were washed with PBS and lysed using a buffer containing 50 mM Tris, 100 mM NaCl, and 0.1% NP40. Lysates were used to assess TG content with the Infinity Triglyceride kit (Thermo Fisher Scientific). TG content was normalized to total protein for each well.

### Glucose tolerance test.

Mice were subjected to an overnight fast, and fasting blood glucose was measured. Glucose in PBS was administered by i.p. injection at a dose of 1.5 mg/kg body weight. Blood glucose concentration was measured at 15, 30, 45, 60, 90, and 120 minutes using a glucometer.

### Histology.

Longitudinal sections of adipose tissue were collected and fixed in Bouin’s solution. Tissue was embedded in paraffin, sectioned, and stained with H&E (performed at the Dana Farber Rodent Histopathology Core Facility). Section images were captured using the EVOS Cell Imaging System (Thermo Fisher Scientific).

### GWAS.

The associations of genetic variants located at *IL13RA1*, *IL13RA2*, and *IL4R* with BMI and T2D were acquired from GWAS summary statistics made available from published studies conducted at the Biobank Japan (for both BMI and T2D) (34, 35), the GIANT Consortium (for BMI) (48), and the Diabetes Genetics Replication and Meta-analysis (DIAGRAM) Consortium (for T2D adjusted for BMI) (49). Regional association plots were generated using LocusZoom (66). Figures of tissue-specific gene expression (in subcutaneous and visceral adipose tissues, liver, and muscle) were generated using the GTEx Portal (50).

### Statistics.

All data are presented as the mean ± SEM. Statistical analyses were performed using GraphPad Prism 7 (GraphPad Software), with the exception of RNA-Seq analysis. A 2-tailed, unpaired *t* test was used for comparison of 2 parameters. One-way ANOVA with Tukey’s multiple-comparison test was used for analysis of in vitro experiments with more than 2 treatment conditions. Two-way ANOVA with Tukey’s multiple-comparison test was used for analysis of in vitro experiments with 2 independent variables. Two-way ANOVA was used for evaluation of cold tolerance tests and GTTs. Most cell-based experiments were repeated 2–4 times. RNA-Seq and validation of RNA-Seq in sh*Il13ra1* and *Il13ra1*-RE cell lines were performed 1–2 times. Cell-based experiments were performed with 3–10 replicates, as noted in the figure legends.

### Study approval.

All animal studies were approved by the Harvard Medical Area Standing Committee on Animal Research and Academia Sinica IACUC (23-10-2066 for *Il4*-KO mouse and neutralizing antibody studies).

### Data availability.

The complete RNA-Seq datasets have been deposited in the Gene Expression Omnibus (GEO) database (GEO GSE171617). Gene lists for ontology analyses of differentiated adipocytes and preadipocytes are available in Supplemental Data Files 1 and 2, respectively. Values for all data points shown in graphs of the main and Supplemental Figures are presented in the Supporting Data Values file.

## Author contributions

ARY, MMC, NHK, ALH, JP, HC, YHL, CWL, and KAS performed the experiments. ALH, NHK, MMC, and ARY contributed to methods optimization and development of reagents. JL and FBH surveyed genetic associations from published GWAS and gene expression in human tissues in the GTEx Portal. ASB oversaw CLAMS studies and provided feedback on data interpretation. JSL provided *Il4*-KO mouse cohorts, and DYT and HWH conducted *Il4*-KO–related experiments. ARY, MMC, and CHL conceptualized the study, designed experiments, and interpreted results. ARY and CHL wrote the manuscript. ARY and MMC are listed as co–first authors because of their shared contributions to the project. ARY wrote and organized the manuscript and is therefore listed first among the 2 co–first authors.

## Supplementary Material

Supplemental data

Supplemental data set 1

Supplemental data set 2

Unedited blot and gel images

Supporting data values

## Version 1 04/08/2025

In-Press Preview

## Version 2 06/02/2025

Electronic publication
