# Supplementary material for: Preadipocyte IL-13/IL-13Rα1 signaling regulates beige adipogenesis through modulation of PPARγ activity
Source: J Clin Invest. 2025 Apr 8;135(11):e169152. doi: 10.1172/JCI169152 (PMC12126228; doi:10.1172/JCI169152)
Supplement: Supplemental data [file jci-135-169152-s154.pdf]

Supplemental Information

Preadipocyte IL-13-IL-13Rα1 signaling regulates beige adipogenesis through modulation of PPARγ activity

Alexandra R. Yesian, Mayer M. Chalom, Nelson H. Knudsen, Alec L. Hyde, Jean Personnaz, Hyunjii Cho, Yae-Huei Liou, Kyle A. Starost, Chia-Wei Lee, Dong-Yan Tsai, Hsing-Wei Ho, Jr-Shiuan Lin,Jun Li, Frank B. Hu, Alexander S. Banks and Chih-Hao Lee

Supplemental Figures

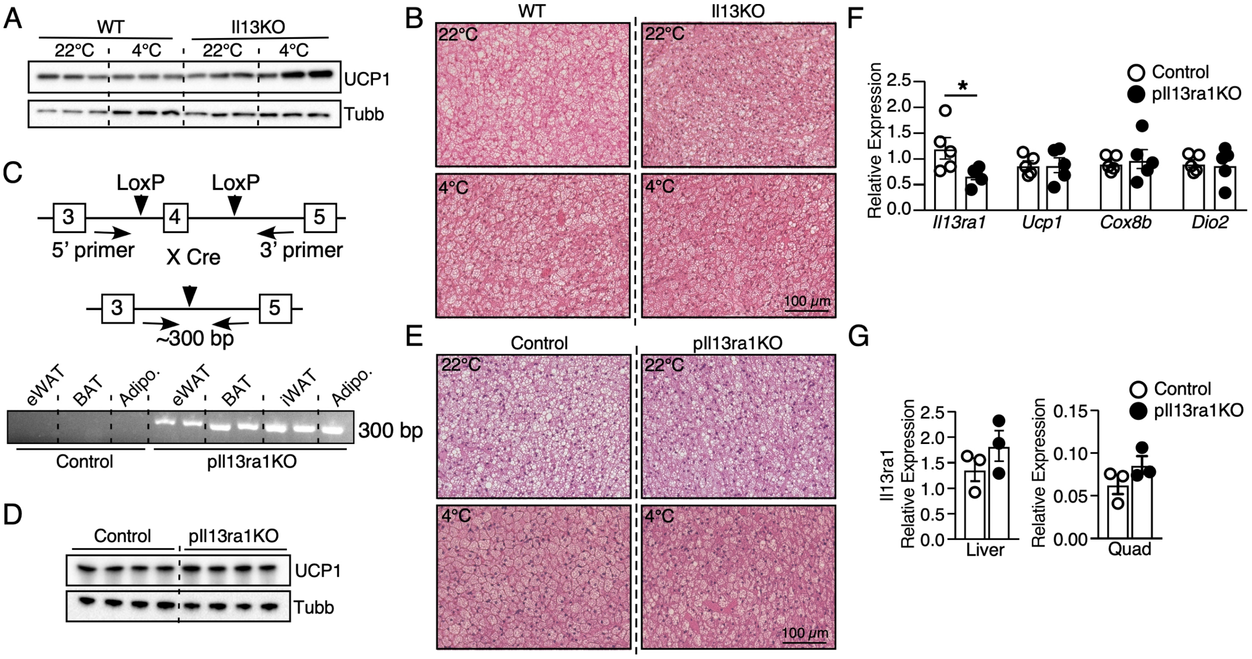

Supplemental Figure 1. IL-13 /IL-13Rα1 signaling does not regulate cold-induced brown adipocyte recruitment. (**A**) Immunoblots showing UCP1 protein in BAT of WT and Il13KO mice housed at 4°C or 22°C for 72 hours. n=5-6 per group, 8-week-old female mice, experiment performed once. Representative tissue samples from 3 mice/group are shown. Tubb: tubulin as a loading control. (**B**) Representative H&E staining images of BAT from mice in (A). (**C**) Schematic of *Il13ra1* deletion site and detection of genomic DNA for the 300 base-pair excised fragment of *Il13ra1* in the adipose depots of control and pIl13ra1KO mice. n=2 mice/group, 5-6-month-old male control mice; 7-week-old male pIl13ra1KO mice, experiment performed once. (**D**) Immunoblotting of UCP1 protein, (**E**) Representative H&E staining images and (**F**) Expression of *Il13ra1* and thermogenic genes measured by RT-qPCR in BAT of control and pIl13ra1KO mice after a 72-hour cold tolerance test at 4°C. n=5/group, 5-7-week-old male mice, experiment performed once. Age-matched room-temperature controls were used in (E). (**G**) Expression of *Il13ra1* in liver and quadriceps (Quad) muscle of control and pIl13ra1KO mice. n=3 mice/group, 6-8-month-old male mice, experiment performed once. Statistical analysis performed using unpaired Student's t-test (to compare control vs pIl13ra1KO). Values are presented as mean ± SEM. *$p<0.05$.

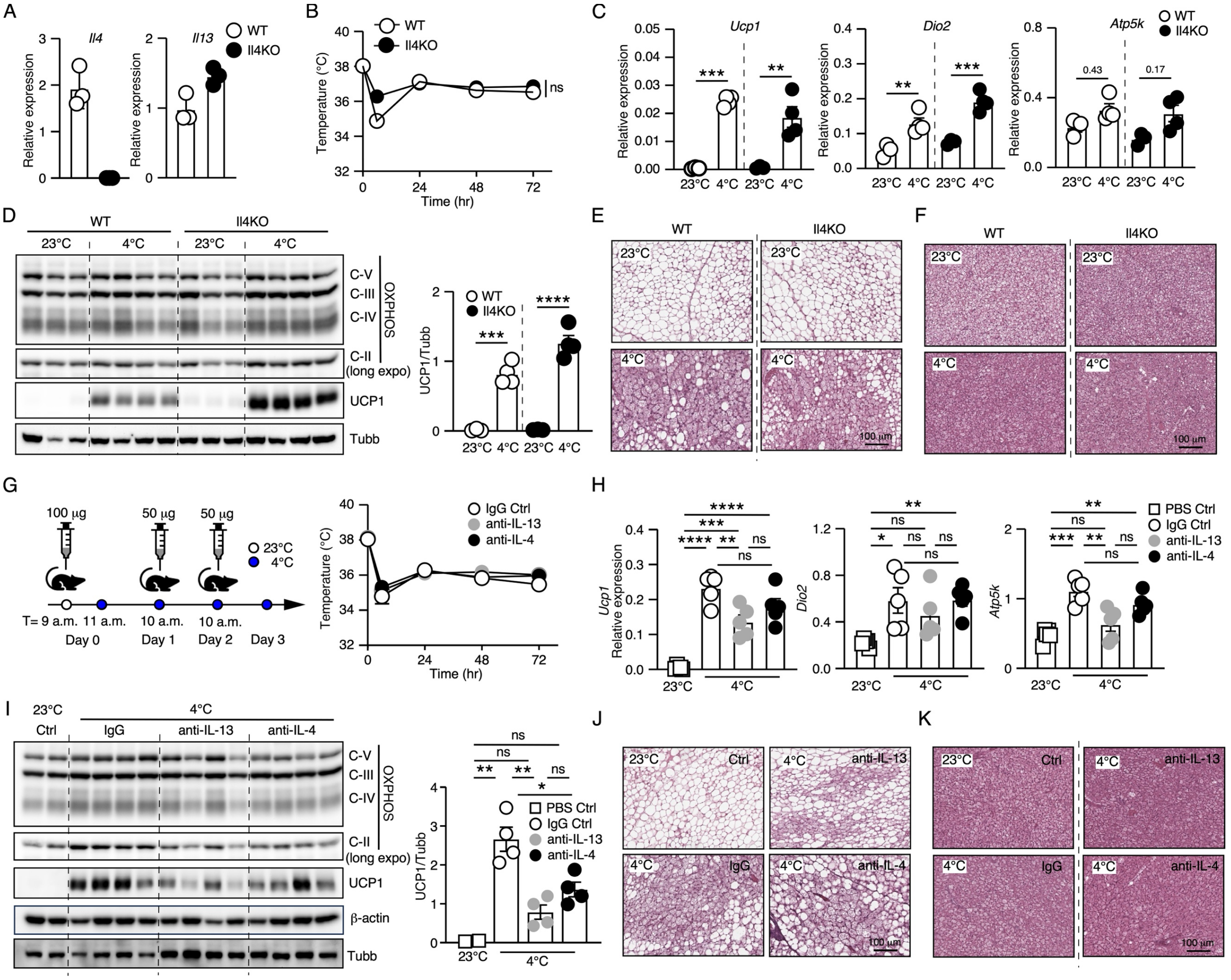

Supplemental Figure 2. IL-4 is not required for cold-induced beige adipocyte recruitment. (**A**) The expression of *Il4* and *Il13* by real-time PCR in splenocytes from WT and Il4KO mice stimulated with phorbol myristate acetate (5 ng/ml) and ionomycin (500 ng/ml) for 4 hours. (**B**) Core body temperature of WT and Il4KO mice during a 72-hour cold tolerance test at 4°C. n=4, 6-8-week-old female mice. Statistical analysis performed using 2-way ANOVA. Experiments in (B)-(F) repeated in one additional female cohort and one male cohort that were 12-week-old. (**C**) Assessing the expression of thermogenic genes in iWAT by real-time PCR. 6-8-week-old female mice, n=3 for room temperature at 23°C; n=4 at 4°C. Statistical analysis performed using unpaired Student's t-test (comparing 23°C vs 4°C of the same genotype). (**D**) Immunoblots showing protein levels of UCP1 and mitochondrial OXPHOS complexes II (SDHB), III (UQCRC2), IV (MTCO1), and V (ATP5A) in iWAT of WT and Il4KO mice. Right panel: UCP1 immunoblot signal normalized to the loading control Tubulin (Tubb). Statistical analysis performed using unpaired Student's t-test (comparing 23°C vs 4°C of the same genotype). (**E**) and (**F**) Representative H&E staining of iWAT and BAT, respectively, from mice in (B)-(D). (**G**) Left: Schematic showing neutralizing antibody treatment performed 2 hours before (at the dose of 100 µg/animal) and 24 and 48 hours (at 50 µg) after initiation of a 72-hour cold exposure. Right: Core body temperature of mice receiving anti-IL-13 antibody, anti-IL-4 antibody or IgG control during a 72-hour cold tolerance test at 4°C. n=5/group, 7-8-week-old female mice. Statistical analysis performed using

2-way ANOVA. Experiment performed in one cohort. (**H**) Assessing the expression of thermogenic genes in iWAT by real-time PCR. The room temperature control mice were injected with PBS (n=5). Statistical analysis performed using 2-way ANOVA with Tukey's multiple comparisons test. (**I**) Immunoblots showing protein levels of UCP1 and mitochondrial OXPHOS complexes V (ATP5A), IV (MTCO1), III (UQCRC2) and II (SDHB) in iWAT from mice in (H). Representative tissue samples from 5 mice/group are shown. Right panel: UCP1 immunoblot signal normalized to the loading control Tubulin (Tubb). Statistical analysis performed using 2-way ANOVA with Tukey's multiple comparisons test. (**J**) and (**K**) Representative H&E staining of iWAT and BAT, respectively, from mice in (H). Values are presented as mean ± SEM. *p<0.05, **p<0.01, ***p<0.001, ****p<0.0001.

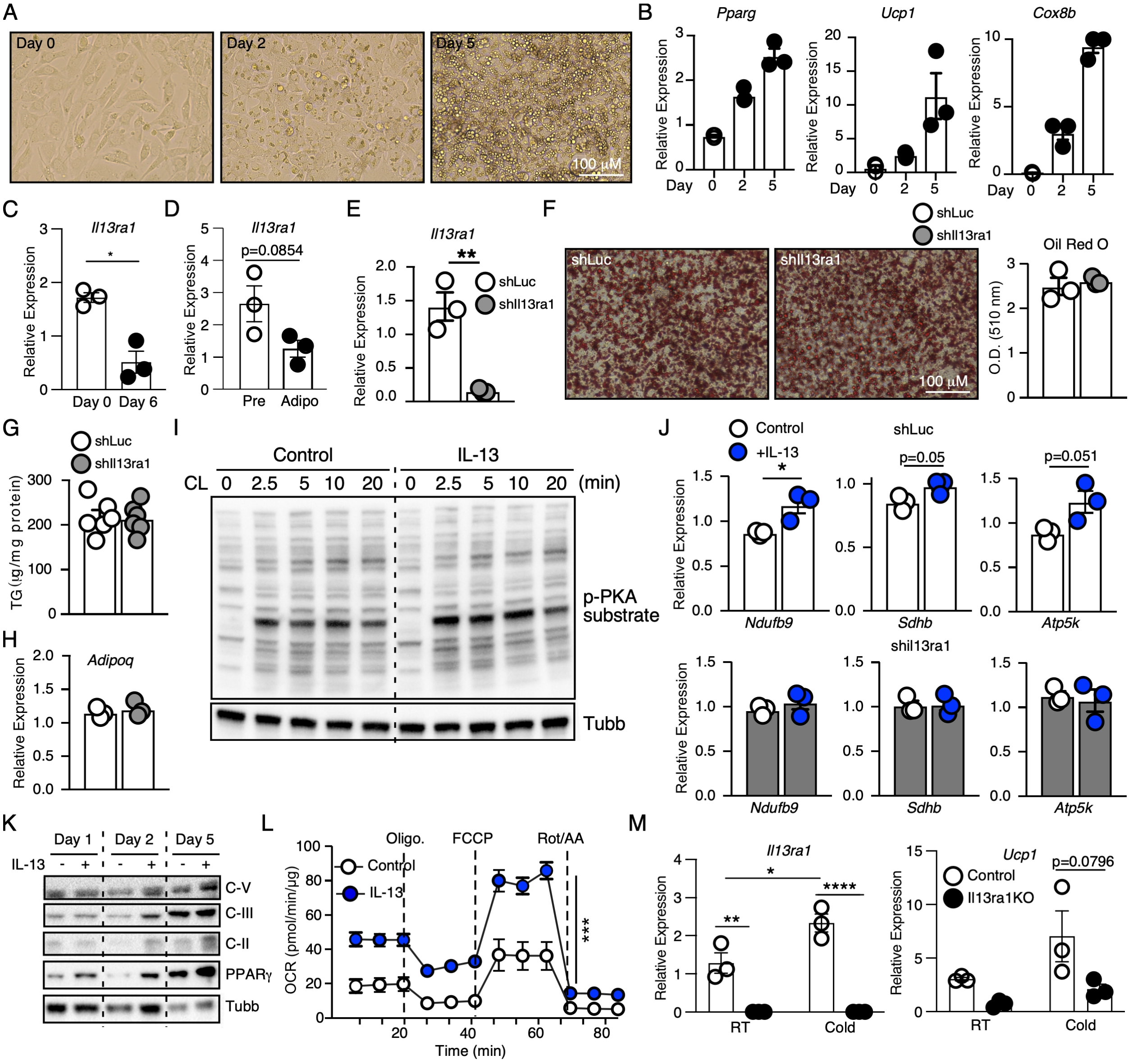

Supplemental Figure 3. Characterization of immortalized preadipocyte cell line for mechanistic studies. (**A**) Representative bright field images of WT B6 clonal cell line (referred to as WT) on Days 0, 2, and 5 of differentiation. (**B**) Expression of adipogenic and thermogenic genes measured by RT-qPCR in WT preadipocyte cell line during differentiation. n=3, experiment performed 3 times. (**C**) Expression of *Il13ra1* in WT preadipocytes before (Day 0) and following (Day 6) differentiation. n=3. Statistical significance analyzed using unpaired Student's t-test. (**D**) Expression of *Il13ra1* in primary preadipocytes (Pre) and primary adipocytes (Adipo) isolated from the iWAT of C57B6/J female mice. n=3. Statistical significance analyzed using unpaired Student's t-test. (**E**) Expression of *Il13ra1* in control (shLuc) and *Il13ra1*-knockdown (shIl13ra1) preadipocytes, n=3, experiment performed 2 times. Statistical significance performed using unpaired Student's t-test. (**F**) Oil Red O staining and quantification of differentiated shLuc and shIl13ra1 adipocytes. n=3, experiment performed once. Statistical analysis performed using unpaired Students t-test. (**G**) TG content (n=6, experiment performed 2 times) and (**H**) Expression

of *Adipoq* by RT-qPCR (n=3, experiment performed 3 times) in differentiated shLuc and shIl13ra1 adipocytes. (**I**) Western blotting time course analysis of phosphorylated PKA substrate in WT adipocytes differentiated with or without IL-13 pre-treatment. Adipocytes were differentiated for 6 days and then treated with CL316243 (CL) to induce PKA activity. n=1 well per timepoint. Experiment repeated twice. (**J**) Expression of OXPHOS genes measured by RT-qPCR in shLuc control and shIl13ra1 adipocytes that were pretreated with/without IL-13 for 24 hours and differentiated for 5 days. n=3, experiment performed once. Statistical significance performed using unpaired Student's t-test. (**K**) Immunoblotting of PPARγ protein and mitochondrial OXPHOS complexes II (SDHB), III (UQCRC2), and V (ATP5A) on Days 0, 2, and 5 of differentiation. WT preadipocytes were pretreated with IL-13 or vehicle control before differentiation. Tubb: tubulin as a loading control. n=1, experiment performed once. (**L**) The oxygen consumption rate (OCR) of WT cells treated with/without IL-13 for 24-hours and differentiated for 2 days measured by Seahorse. Oligomycin (Oligo) was used to inhibit coupled respiration. FCCP was added to measure maximal respiration. Rotenone and antimycin A (Rot/AA) were added to inhibit mitochondrial respiration. n=5, experiment performed twice. (**M**) Expression of *Il13ra1* and *Ucp1* in differentiated primary preadipocytes isolated from room temperature (RT) and cold-exposed WT or Il13ra1KO mice. Cold exposure was performed at 4°C for 72 hours. Primary preadipocytes were harvested from iWAT and differentiated for six days *ex vivo*. n=3. Experiment performed one time. Statistical significance performed using 2-way ANOVA. Values are presented as mean ± SEM. *p<0.05, **p<0.01, ***p<0.001.

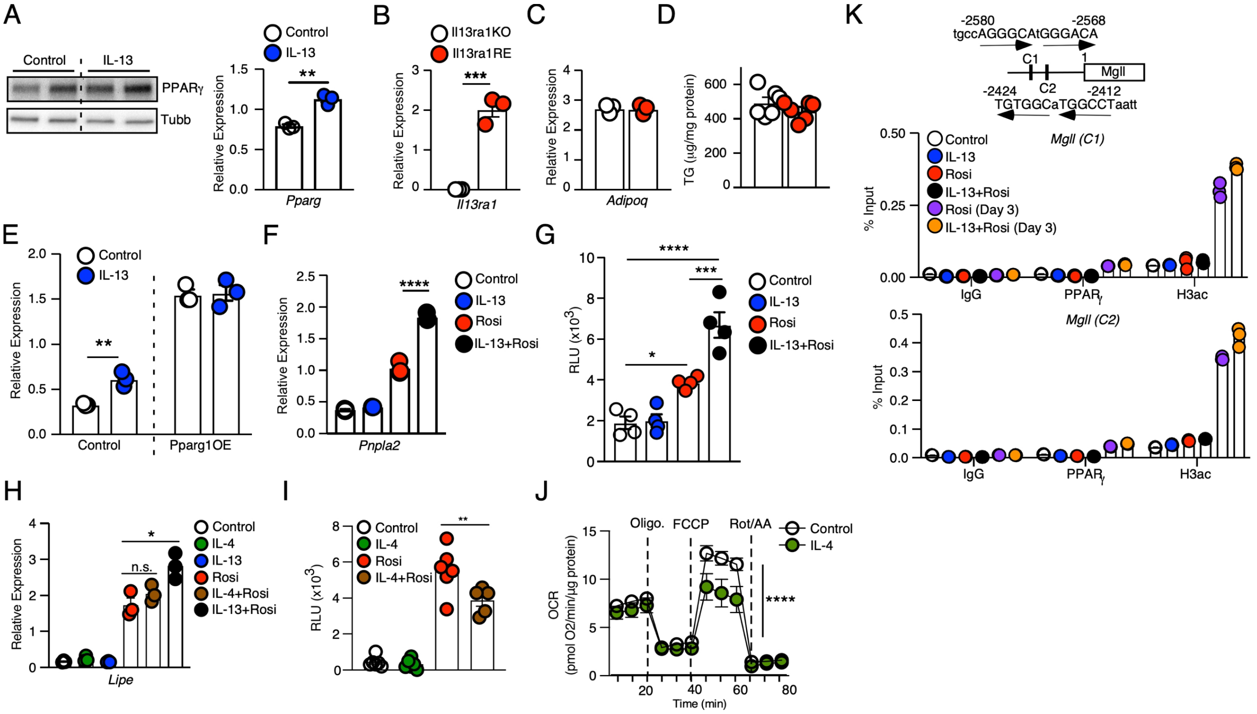

Supplemental Figure 4. *Pparg* is a direct target of IL-13 in preadipocytes. (**A**) Immunoblotting of PPARγ protein (left, n=2) and expression of *Pparg* measured by RT-qPCR (right, n=3) in primary SVF isolated from 8-week-old C57BL/6J mice (see Figure 2J). Cells were treated with vehicle control or IL-13 for 24 hours, experiments performed once. Tubb: tubulin as a loading control. (**B**) *Il13ra1*expression measured by RT-qPCR in *Il13ra1*-knockout (Il13ra1KO, with pBabe empty vector as the control) and *Il13ra1*-reexpression (Il13ra1RE) preadipocytes. n=3, experiment performed 3 times. Statistical significance performed using unpaired Student's t-test. (**C**) *Adipoq* expression by RT-qPCR (n=3, experiment performed 2 times) and (**D**) TG content (n=6) in differentiated Il13ra1KO and Il13ra1RE adipocytes. n=3, experiment performed 2 times. Statistical significance performed using unpaired Student's t-test. (**E**) *Pparg* expression by RT-qPCR in Control (pBabe) and *Pparg1*-overexpressing (Pparg1OE) preadipocytes treated with/without IL-13 for 24 hours. n=3, experiment performed 2 times. Statistical analysis performed using unpaired Student's t-test (comparing ±IL-13 within the group). **F**) *Pnpla2* expression measured by RT-qPCR in Pparg1OE preadipocytes treated with control, IL-13, rosi, or IL-13+rosi for 24 hours. n=3, experiment performed once. Statistical analysis performed using one-way ANOVA with Tukey's multiple comparisons test. (**G**) Quantification of PPRE reporter activity in Pparg1OE preadipocytes treated with control, IL-13, rosi, or IL-13+rosi for 24 hours. The PPAR response element (PPRE) containing luciferase reporter was co-transfected with a β-galactosidase internal control. Luciferase activity was measured 48 hours after transfection and normalized to β-galactosidase activity to determine the relative luciferase unit (RLU). n=4, experiment performed 3 times. Statistical analysis performed using 2-way ANOVA with Tukey's multiple comparisons test. **(H)** *Lipe* gene expression in WT preadipocytes treated for 24 hours with vehicle, IL-4, or IL-13 ± rosi. n=3. Statistical analysis performed using one-way ANOVA with Tukey's multiple comparisons test. (**I**) Quantification of PPARγ-LBD transactivation activity on the luciferase reporter in WT preadipocytes treated with control, IL-4, rosi, or IL-4+rosi for 24 hours. Luciferase activity was measured 48 hours after transfection and normalized to β-galactosidase activity to

determine the relative luciferase unit (RLU). n=6, with one outlier excluded from analysis. Statistical analysis performed using one-way ANOVA with Tukey's multiple comparisons test. (**J**) Mitochondrial respiration (determined by the oxygen consumption rate, OCR) of WT preadipocytes treated with vehicle or IL-4 for 24-hours. n=5. Statistical significance performed using 2-way ANOVA. (**K**) Upper panel: Two sets of ChIP primer pairs (C1 and C2) were designed around two potential PPARγ binding sites (PPREs) in the 5' regulatory region of the *Mgll* gene. The transcriptional start site is designated as 1. The arrows indicate two direct repeat motifs of the PPRE. Lower panel: Chromatin immunoprecipitation was performed on preadipocytes treated overnight with IL-13, rosi, or IL-13+rosi and on adipocytes differentiated for three days after overnight rosi or IL-13+rosi pretreatment. Protein-DNA complexes were pulled down using antibodies for IgG control, PPARγ or acetylated histone H3 (H3ac, H3K27ac). Real-time quantitative PCR was performed using C1 and C2 primer pairs. n=3 technical replicates per condition, experiment was performed 3 times. Values are presented as mean ± SEM. *p<0.05, **p<0.01, ***p<0.001, ****p<0.0001.

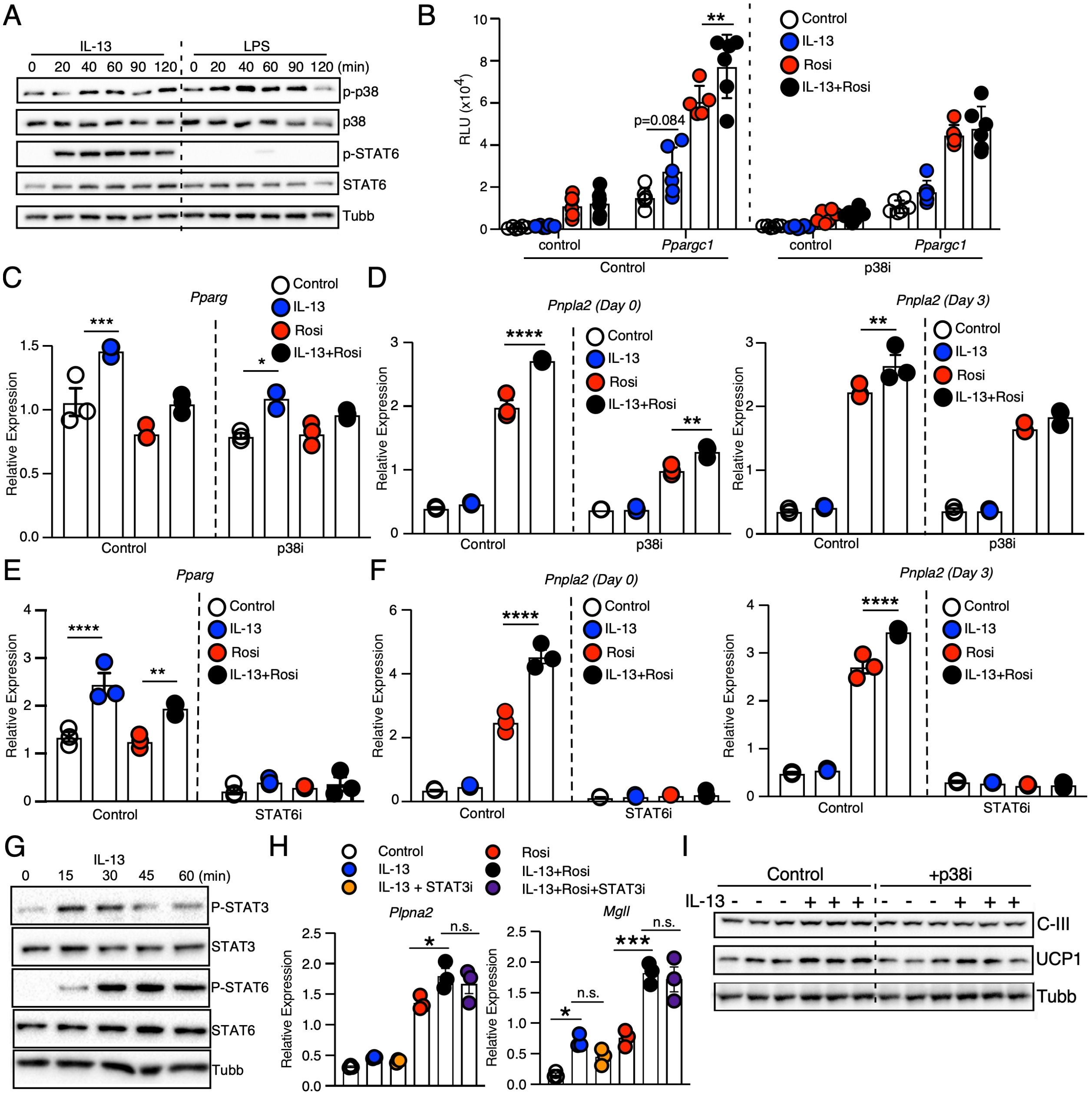

Supplemental Figure 5. IL-13/IL-13Rα1 activates p38 MAPK and STAT6 to modulate PPARγ activity. (**A**) Immunoblot analysis of STAT6, phospho-STAT6 (p-STAT6), p38-MAPK, and phospho-p38-MAPK (p-p38) in preadipocytes treated IL-13 or LPS for 0-120 minutes. n=1 per timepoint, experiment performed 2 times. LPS serves as a control for p38 activation. Tubb: tubulin as a loading control. (**B**) Quantification of PPARγ-LBD transactivation activity in WT preadipocytes co-transfected with the luciferase reporter, Gal4-PPARγ-LBD and control vector or *Ppargc1a* expression vector. Cells were treated with vehicle control, IL-13, rosi, or IL-13+rosi for 24 hours ± P38 inhibitor (P38i). Luciferase activity was measured 48 hours after transfection and normalized to protein content to determine the relative luciferase unit (RLU). n=6, experiment performed 3 times. Statistical analysis performed using 2-way ANOVA with Tukey's multiple comparisons test. (**C**) *Pparg* expression measured by RT-qPCR in WT preadipocytes treated with

vehicle control, IL-13, rosi, or IL-13+rosi for 24 hours with/without P38i. n=3, experiment performed twice with P38i. Statistical analysis performed using 2-way ANOVA with Tukey's multiple comparisons test. (**D**) Expression of the PPARγ target gene *Pnpla2* measured by RT-qPCR in WT preadipocytes treated with vehicle control, IL-13, rosi, or IL-13+rosi ± P38i for 24 hours. *Pnpla2* expression was determined right after the 24-hour treatment (Day 0) or following 3 days of differentiation (Day 3). n=3, experiment performed 2 times for Day 0, once for Day 3. Statistical analysis performed using 2-way ANOVA with Tukey's multiple comparisons test. (**E**) *Pparg* expression measured by RT-qPCR in WT preadipocytes treated with vehicle control, IL-13, rosi, or IL-13+rosi for 24 hours with/without STAT6i. n=3, experiment performed once. Statistical analysis performed using 2-way ANOVA with Tukey's multiple comparisons test. (**F**) *Pnpla2* expression measured by RT-qPCR in WT preadipocytes right after the 24-hour treatment (Day 0) or following 3 days of differentiation (Day 3). Statistical analysis performed using 2-way ANOVA with Tukey's multiple comparisons test. n=3, experiments performed once. (**G**) Immunoblotting of STAT3 and STAT6 phosphorylation in WT preadipocytes treated with IL-13 for 0, 15, 30, 45, or 60 minutes. Experiment performed once. (**H**) Gene expression from WT preadipocytes treated 24 hours with vehicle control, IL-13, rosi, IL-13+rosi ± STAT3 inhibitor (STAT3i) for 24 hours. n=3. Statistical analysis performed using 1-way ANOVA with Tukey's multiple comparisons test. (**I**) Immunoblotting showing complex III (UQCRC2) and UCP1 protein levels in differentiated adipocytes. Preadipocytes were pretreated with rosi plus IL-13 or vehicle ± p38i for 24 hours, followed by differentiation for 5 days. n=3, experiment performed once. Values are presented as mean ± SEM. *p<0.05, **p<0.01, ***p<0.001, ****p<0.0001.

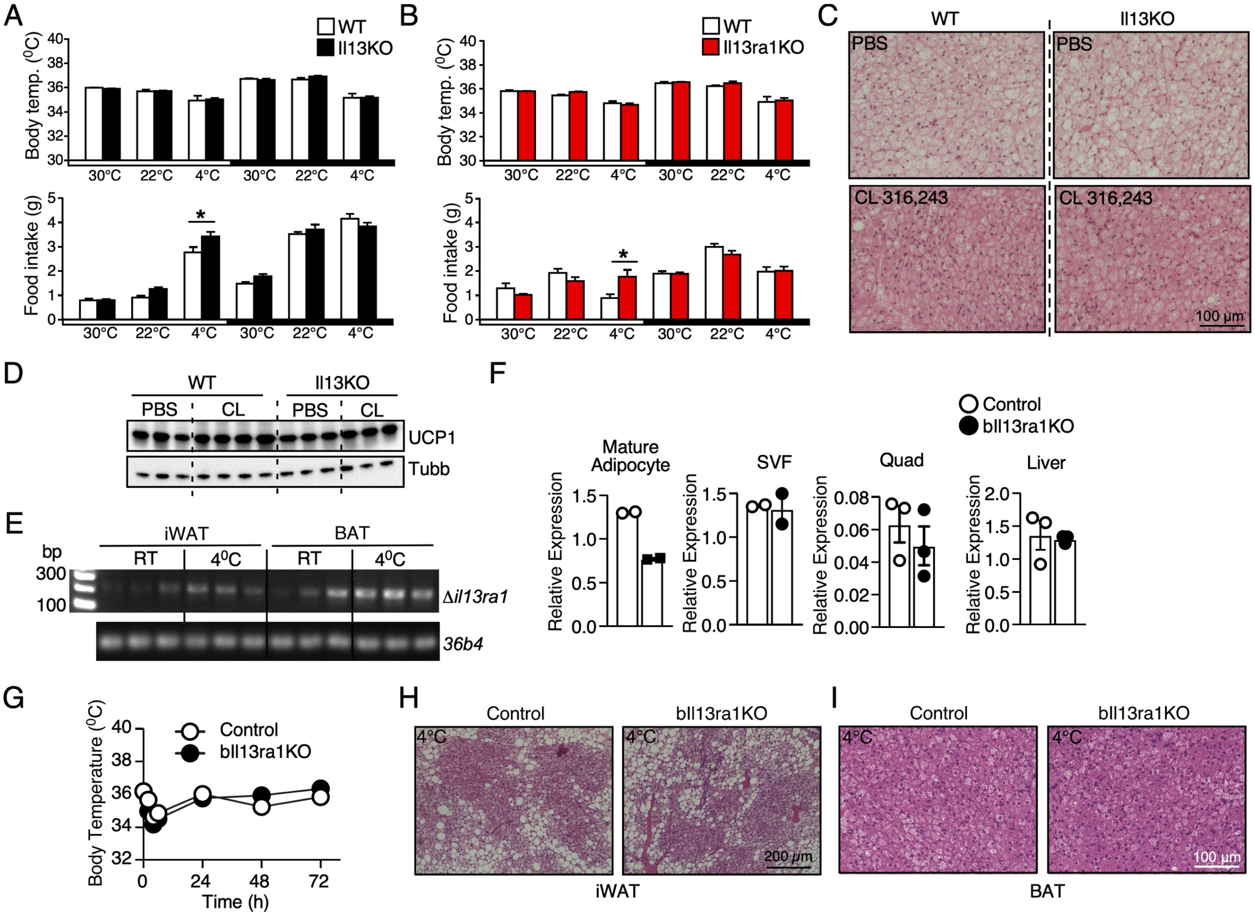

Supplemental Figure 6. Intact brown adipose tissue function in mice lacking IL-13 signaling. (**A**) Core body temperature and food intake of WT and Il13KO mice housed at 30°C, 22°C and 4°C in metabolic cages for 72 hrs. Data presented as average temperature for light and dark cycles after 48 hours acclimation at each temperature. n=6, 20-week-old male mice, experiment performed once. Statistical analysis performed with unpaired Student's t-test (comparing WT and Il13KO). (**B**) Core body temperature and food intake of WT and Il13ra1KO mice housed at 30°C, 22°C and 4°C for 72 hrs. Data presented as average temperature for light and dark cycles after 48 hours acclimation at each temperature. n=5-7 per group, 24-week-old male mice, experiment performed once. Statistical analysis performed with unpaired Student's t-test (comparing WT and Il13ra1KO). (**C**) Representative H&E staining images of BAT and (**D**) Immunoblotting showing UCP1 protein in BAT from WT and Il13KO mice injected with PBS or CL for 10 days. n=3-4 per group, 3-month-old males, experiment performed once. Tubb: tubulin as a loading control. (**E**) Genomic DNA validation of *Il13ra1* gene deletion (~300 base-pair excised fragment) by qPCR in iWAT and BAT from iWAT of bIl13ra1KO mice housed at room temperature (RT) or 4°C. n=3 mice/group; 3-month-old male mice. Products of qPCR were visualized on an agarose gel. Experiment performed once. (**F**) *Il13ra1* expression measured by RT-qPCR in mature primary adipocytes and SVF from iWAT, quadriceps muscle and liver of control and bIl13ra1KO mice. For quad and liver n=3, 6-8-month-old male mice, experiment performed once; n=2 replicates for primary adipocytes and SVF pooled from 3 mice per genotype, 5-6-month-old male mice. Experiments performed once. (**G**) Core body temperature of control and bIl13ra1KO mice during

a 72-hour cold tolerance test at 4°C. n=6, 6-week-old male mice, experiment performed twice. Statistical analysis performed using 2-way ANOVA. (**H**) and (**I**) Representative H&E staining images of iWAT and BAT from mice in (G). Values are presented as mean ± SEM. *$p<0.05$.

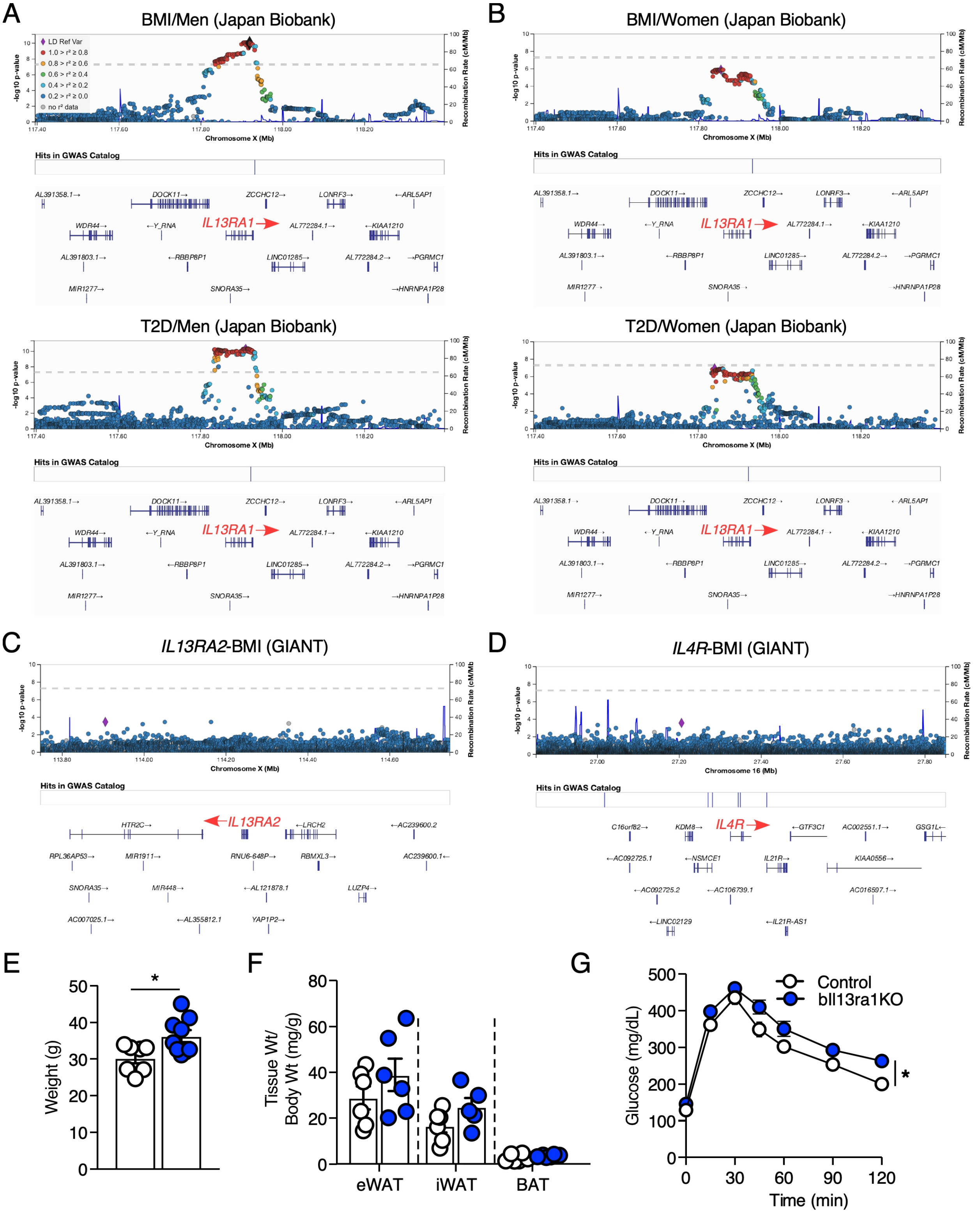

Supplemental Figure 7. Association of genetic variants at *IL13RA1* with body mass index (BMI) and type 2 diabetes (T2D). (**A**) and (**B**) *IL13RA1* genetic variants are associated with BMI and T2D in a Japanese population (data acquired from BioBank Japan based on published GWAS,

see Methods). The regional association plot was generated by Locuszoom (https://my.locuszoom.org/) on -log10 p values for each genetic variant's association with the trait. Each dot is a genetic variant, and the diamond shaped dot indicates the lead variant (i.e., variant with the smallest p value). Colors of the dots indicate LD relationship (r^2) between all genetic variants to the lead variants. Minor allele frequency (MAF) was not included. (**C**) and (**D**) No genome-wide significant association with BMI at the *IL13RA2* and *IL4R* loci, based on published GWAS summary data from a GIANT Consortium study (see Methods). (**E**) Body weight and (**F**) Fat tissue weight normalized to body weight of Control and bIl13ra1KO mice. n=7-9/group, 4-month-old males. Statistical significance performed using unpaired Student's t-test. Experiment repeated in two separate cohorts. (**G**) GTT of Control and bIl13ra1KO mice. n=6 Control, n=10 bIl13ra1KO, 3-month-old males, experiment repeated in 3 separate cohorts. Statistical significance determined with 2-way ANOVA. Values are presented as mean ± SEM. *$p<0.05$.

Supplemental Table 1. Animal Cohorts

Experiment	Genotype/Number of Mice	M/F	Age	Figures
WT vs. Il13KO 72-hour cold exposure	WT: n=6 22°C, n=6 4°C; Il13KO: n=5 22°C, n=5 4°C	Female	8 weeks	Fig. 1A-C Fig. S1A-B Fig. 2K
Flox control vs. pAdIl13ra1KO 72-hour cold exposure	Control: n=5 4°C pIl13ra1KO: n=5 4°C	Male	5-7 weeks	Fig. 1D-G Fig. S1C-E Fig. 6F-H
WT vs. Il4KO 72-hour cold exposure	WT: n=3 23°C, n=4 4°C; Il4KO: n=3 23°C, n=4 4°C	Female	6-8 weeks	Fig. S2A-F
PBS injected vs neutralizing antibodies injected 72-hour cold exposure	Control: n=5 PBS 23°C IgG control: n=5 4°C Anti-IL-13: n=5 4°C Anti-IL-4: n=5 4°C	Female	7-8 weeks	Fig. S2G-K
WT vs. Il13KO 10-day CL injection	WT: n=5 PBS, n=5 CL Il13KO: n=3 PBS, n=4 CL	Male	12 weeks	Fig. 5A-B Fig. S6C-D
WT vs. Il13ra1KO CL injection	WT: n=4 Il13ra1KO: n=4	Male	30 weeks	Fig. 5C-D
Flox control vs. bIl13ra1KO 7-day CL injection	Control: n=3 non-injected, n=7 CL bIl13ra1KO: n=3 non-injected, n=6 CL	Female	20 weeks	Fig. 5E-F
WT vs. Il13KO adult temperature and food intake (metabolic cages)	n=5-7/group	Male	20 weeks	Fig. S6A
WT vs. Il13ra1KO adult temperature and food intake (metabolic cages)	n=5-7/group	Male	24 weeks	Fig. S6B
Flox control vs. bIl13ra1KO 72-hour cold exposure	Control: n=6 4°C bIl13ra1KO: n=6 4°C	Male	6 weeks	Fig. S6G-I
WT vs. Il13ra1KO Body/Tissue Weights/GTT	WT: n=5 Il13ra1KO: n=6	Male	20 weeks	Fig. 6C-E
Flox control vs. bIl13ra1KO Body/Tissue Weights/GTT	GTT: n=6 control, n=9 bIl13ra1KO Weights: n=6 control, n=6 bIl13ra1KO	Male	GTT 12 weeks; weights 16 weeks	Fig. S7E-G
Flox control vs. pIl13ra1KO GTT	Control: n=7 pIl13ra1KO: n=7	Female	20 weeks	Fig.6H

Supplemental Table 2. List of Primer Sequences for qPCR

Gene	Primer Bank ID	Forward Sequence	Reverse Sequence
36b4	N/A	AGATGCAGCAGATCCGCAT	GTTCTTGCCCATCAGCACC
Il13ra1	N/A	CCCTGAAGGTGATCCTGAGT	TAGTGTGTGTCAGGGCTTGT
Ucp1	N/A	GGCCCTTGTAAACAACAAAATAC	GGCAACAAGAGCTGACAGTAAAT
Cox8b	N/A	GAACCATGAAGCCAACGACT	GCGAAGTTCACAGTGGTTCC
Dio2	N/A	CATGCTGACCTCAGAAGGGC	CCAGTTTAACCTGTTTGTAGGCA
Ndufb9	N/A	GGCATCCCTCTGAGAAAGCA	GCTTAACCTCCCGATCCCAG
Sdhb	N/A	TGCAGTTTCAGGCCTGTCGAG	AGGTCCGCACTTATTCAGATCCA
Atp5k*	6671592a1	GTTCAGGTCTCTCCACTCATCA	CGGGGTTTTAGGTAACTGTAGC
Ndufs5	N/A	GCAAGATAGAGTTCGATGACTTCGA	TCTCCCGCTGTTTCTTGAATCA
Pparg*	187960104c1	GGAAGACCACTCGCATTCCTT	GTAATCAGCAACCATTGGGTCA
Adipoq*	87252710c2	GAAGCCGCTTATGTGTATCGC	GAATGGGTACATTGGGAACAGT
Pnpla2	N/A	CCTTAGGAGGAATGCCCTGC	AGCATGTTGGAAAGGGTGGT
Mgll*	6754690a1	CGGACTTCCAAGTTTTTGTCAGA	GCAGCCACTAGGATGGAGATG
Lipe	N/A	ACACAAAGGCTGCTTCTACG	CTCGTTGCGTTTGTAGTGCT
Abcd2*	6752942a1	ATACACATGCTAAATGCAGCAGC	GCCAATGATGGGATAGAGGGT
Pparg1	N/A	CAGGAGCCTGTGAGACCAAC	ACCGCTTCTTTCAAATCTTGTCTG
Bscl2*	236465188c2	TCCTTCTACTACTCCTACATGCC	GGCGGTGGAGGAATCACAG
Cebpa*	6680916a1	CAAGAACAGCAACGAGTACCG	GTCACTGGTCAACTCCAGCAC
Lpl*	6678710a1	GGGAGTTTGGCTCCAGAGTTT	TGTGTCTTCAGGGGTCCTTAG
Adrb3*	133891719c3	CCTTGGGCGAAACTGGTTG	GTTGGTGACAGCTAGGTAGCG
Lipe (C1)	PPRE ChIP	GGCGGTAAATTACCACACG	GCACAACTAAATGACTGGCC
Lipe (C2)	PPRE ChIP	CATAGGACCCAGTCCTGACAG	CTGTCCTCGGAGAAACCTCTC
Mgll (C1)	PPRE ChIP	GCTGTGAGATAATTCACTTCC	GCTGAGGGAGAAACTGCTTTC
Mgll (C2)	PPRE ChIP	CACTGCACACAAATGAGACG	CAAATCCACCGGGGAAAGAC

*Primer sequences were acquired from the MGH Primer Bank

Supplemental Table 3. List of Antibodies and Key Reagents

Type	Designation	Vendor	Catalog #
Antibody	Rabbit monoclonal anti-β-Tubulin	Cell Signaling Technology	2146
Antibody	Rabbit polyclonal anti-UCP-1	Abcam	ab23841; ab209483
Antibody	Mouse anti-Total OXPHOS Cocktail	Abcam	ab110413
Antibody	Mouse monoclonal anti-PPARγ (E-8)	Santa Cruz Biotechnology	sc-7273
Antibody (ChIP)	Rabbit monoclonal anti-PPARγ (81B8)	Cell Signaling Technology	2443
Antibody (ChIP)	Rabbit monoclonal anti-acetyl-Histone H3 (Lys27) (D5E4)	Cell Signaling Technology	8173
Antibody	Rabbit polyclonal anti-β3 adrenergic receptor	Abcam	ab94506
Antibody	Rabbit polyclonal anti-HSL	Cell Signaling Technology	4107
Antibody	Rabbit polyclonal anti-p38-MAPK	Cell Signaling Technology	9212
Antibody	Rabbit polyclonal anti-Phospho-p38 MAPK (Thr180/Tyr182) (3D7)	Cell Signaling Technology	9215
Antibody	Rabbit polyclonal anti-STAT6	Cell Signaling Technology	9362
Antibody	Rabbit polyclonal anti-Phospho-STAT6 (Tyr641)	Cell Signaling Technology	9361
Antibody	Rabbit monoclonal anti-STAT3 (D3Z2G)	Cell Signaling Technology	12640
Antibody	Rabbit monoclonal anti-Phospho-STAT3 (Tyr705) (D3A7)	Cell Signaling Technology	9145
Antibody	Rabbit polyclonal anti-Phospho-HSL (S660)	Cell Signaling Technology	45804
Antibody	Rabbit monoclonal anti-Phospho-PKA Substrate (RRXS*/T*) (100G7E)	Cell Signaling Technology	9624
Antibody	Rabbit monoclonal anti-Hsp60 (EP1006Y)	Abcam	Ab45134
Antibody	*InVivo*MAb mouse IgG1 isotype control (MOPC-21)	BioXCell	BE0083
Antibody	*InVivo*MAb anti-mouse IL-4, (11B11)	BioXCell	BE0045
Antibody	Anti-mIL-13-mIgG1 InvivoFit™ (8H8)	InvivoGen	mil13-mab9-1
Recombinant protein	Recombinant mouse IL-13 protein	R&D Systems	413-ML
Recombinant protein	Lipopolysaccharides from *Escherichia coli* K-235	Sigma-Aldrich	L2143
Recombinant protein	Insulin from bovine pancreas	Sigma-Aldrich	I6634

Recombinant DNA Reagent	pBABE-puro retroviral expression vector (plasmid)	Addgene	Plasmid #1764
Recombinant DNA Reagent	pBABE-Il13ra1 (plasmid)		
Recombinant DNA Reagent	pBABE-Pparg1 (plasmid)		
shRNA	shIl13ra1 forward sequence for plasmid generation: GACGGATCCCTGGAAGAAGTATGACATCTATTCAAGAGATAGATGTCATACTTCTTCCAGTTTTTTGATATCGAATTCGCC		
shRNA	shIl13ra1 reverse sequence for plasmid generation: GGCGAATTCGATATCAAAAAACTGGAAGAAGTATGACATCTATCTCTTGAATAGATGTCATACTTCTTCCAGGGATCCGTC		
Chemical compound	3-Isobutyl-1-methylxanthine (IBMX)	Sigma-Aldrich	I5879
Chemical compound	Dexamethasone	Sigma-Aldrich	D4902
Chemical compound	Rosiglitazone	Sigma-Aldrich	R2408
Chemical compound	CL 316,243	Tocris	1499
Chemical compound	Oligomycin	Abcam	ab141829
Chemical compound	FCCP	Cayman Chemical Company	15218
Chemical compound	Rotenone	Abcam	ab143145
Chemical compound	Antimycin A	Sigma-Aldrich	A8674
Chemical compound	p38-MAPK inhibitor (SB 203580)	Abcam	ab120162
Chemical compound	STAT6 inhibitor (AS1517499)	Selleck Chemicals	S8685
Chemical compound	STAT3 inhibitor (Stattic)	Cayman	14590

Chemical compound	Seahorse XF24 FluxPak	Agilent	102070-00
Assay kit	Infinity^{TM} Triglycerides Liquid Stable Reagent	ThermoFisher Scientific	TR22421
Assay kit	Glycerol Assay Kit	Sigma-Aldrich	MAK117
Assay kit	BCA Protein Assay Kit	Pierce	23223, 1859078
Assay kit	Luciferase Activity Assay	Promega	E1501
Assay kit	SimpleChIP Plus Kit (magnetic beads)	Cell Signaling Technology	9005
Liquid medium	Dulcecco's Modified Eagle's Medium, 4.5g/L glucose	Corning	10-013- CV
Liquid medium	FBS	Gibco	10437-028
Liquid medium	FB Essence	VWR	3100-500
Cell line	AD293 (human embryonic kidney epithelial cells)	Agilent	240085
Cell line	Phoenix-AMPHO (human kidney epithelial cells)	ATCC	CRL-3213
Mouse strain	C57BL6/J	Jackson Laboratory	000664
Mouse diet	PicoLab® Mouse Diet 20	PicoLab	5058
Other	Verso cDNA synthesis kit	ThermoFisher Scientific	AB1453B
Other	NucleoSpin RNA Plus kit	Macherey-Nagel	740984
Other	GeneJET Genomic DNA Purification Kit	ThermoFisher Scientific	K0721
Other	Collagenase, Type II, powder	Gibco	17101-015
Other	Bovine serum albumin	Gemini Bio-Products	700-107P
Other	TranslT-LT1 Transfection Reagent	Mirus Bio	MIR 2300

Supplemental Dataset 1 (separated file). List of differentially expressed genes ($p<0.01$) of differentiated adipocytes identified by RNA-seq comparing IL-13 priming prior to differentiation vs control. n=4.

Supplemental Dataset 2 (separated file). List of differentially expressed genes (FDR<0.01) of preadipocytes identified by RNA-seq comparing IL-13 treatment (24 hours) vs control. n=4.
