# Supplementary material for: Preadipocyte IL-13/IL-13Rα1 signaling regulates beige adipogenesis through modulation of PPARγ activity
Source: J Clin Invest. 2025 Apr 8;135(11):e169152. doi: 10.1172/JCI169152 (PMC12126228; doi:10.1172/JCI169152)

Immunoblot images

Figure 1B

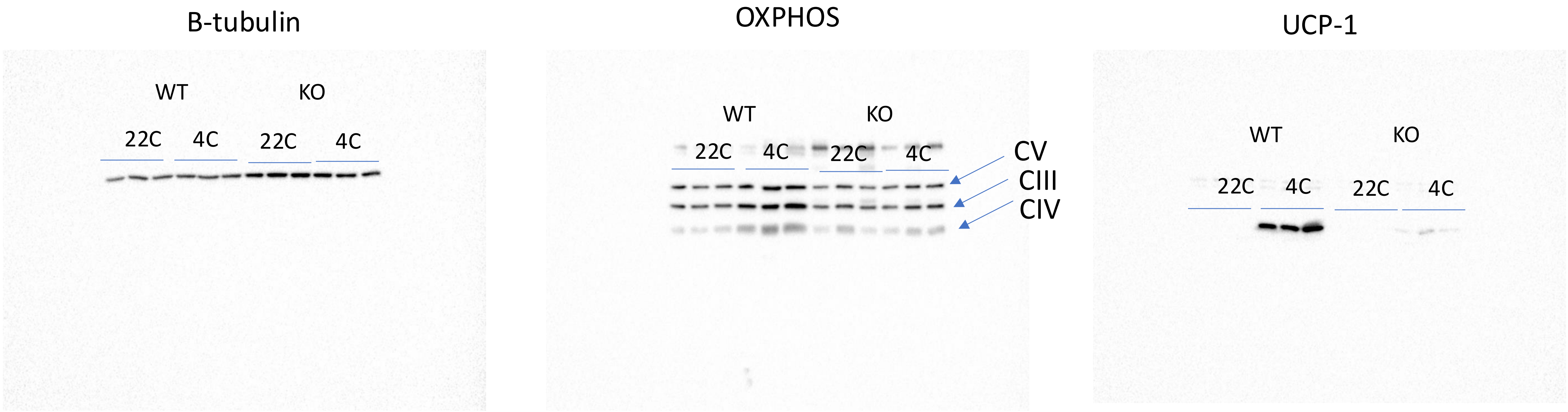
B-tubulin
WT
KO
22C
4C
22C
4C
OXPHOS
WT
KO
22C
4C
22C
4C
CV
CIII
CIV
UCP-1
WT
KO
22C
4C
22C
4C

Figure 1E

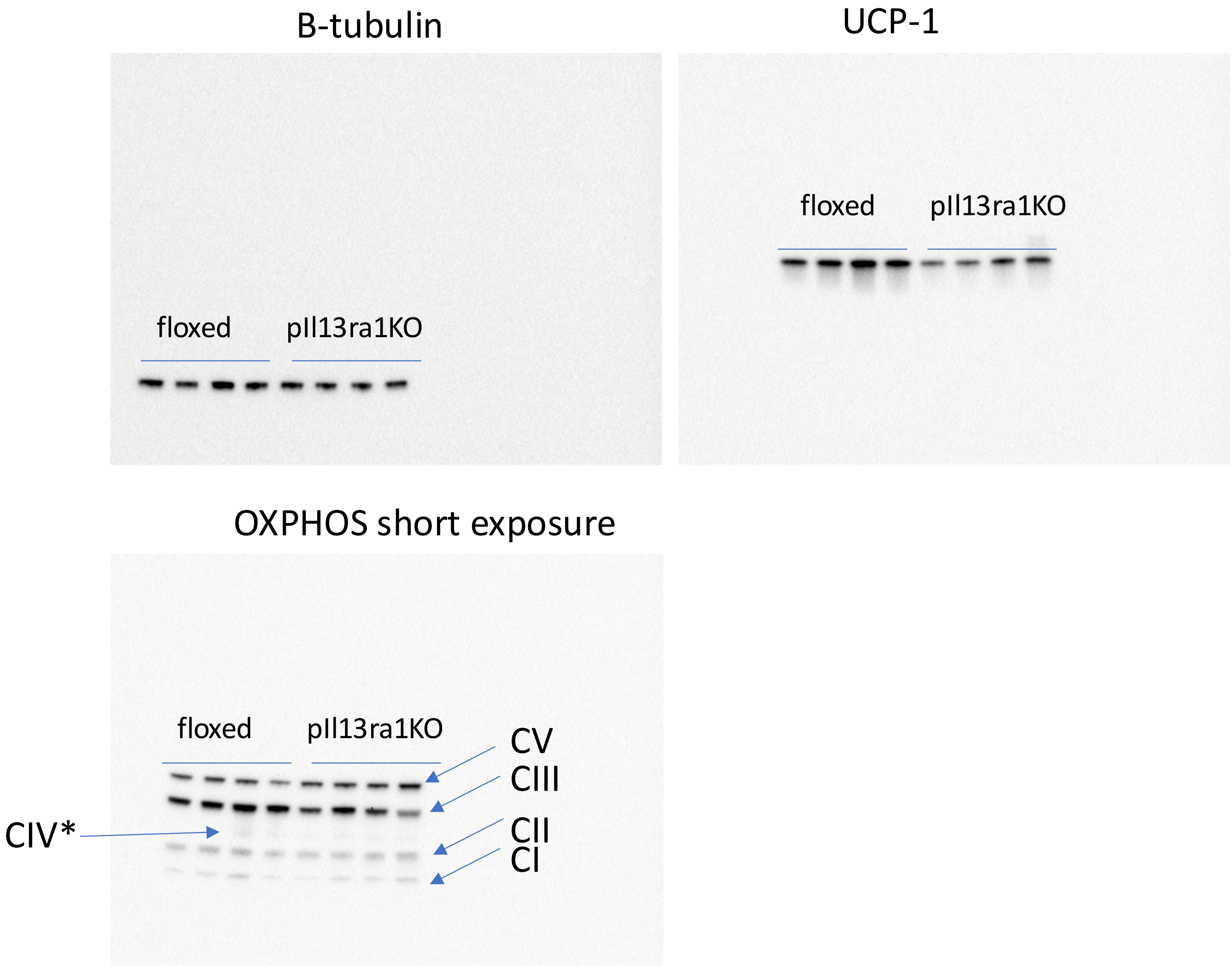
B-tubulin
floxed
pIl13ra1KO
UCP-1
floxed
pIl13ra1KO
OXPHOS short exposure
floxed
pIl13ra1KO
CV
CIII
CIV*
CII
CI

Supplemental Figure 1A

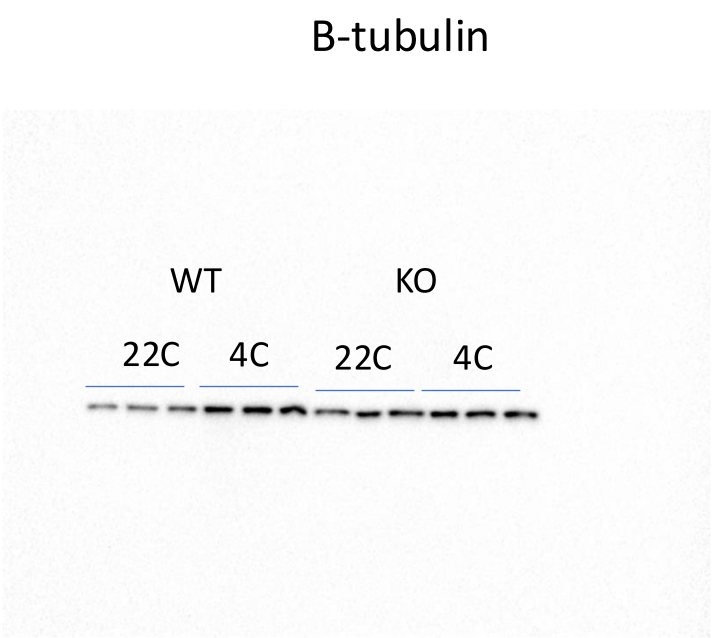

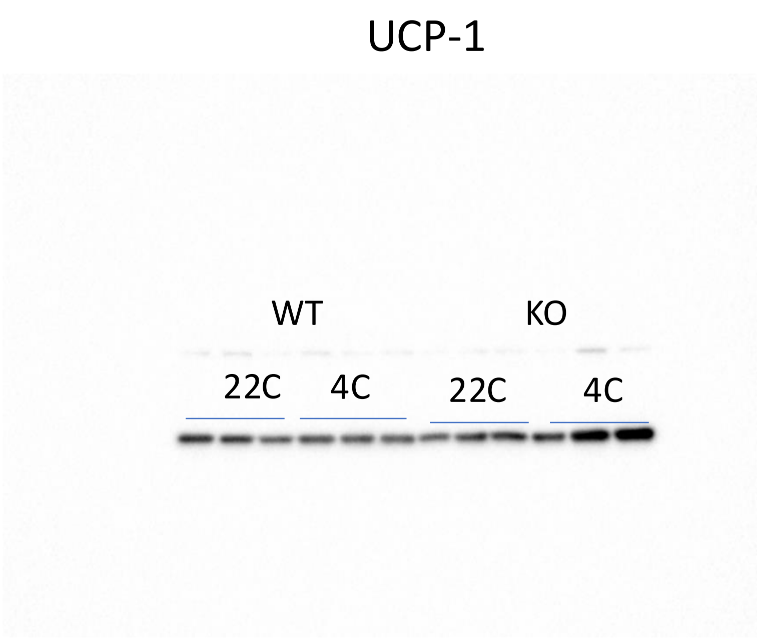

Supplemental Figure 1D

B-tubulin

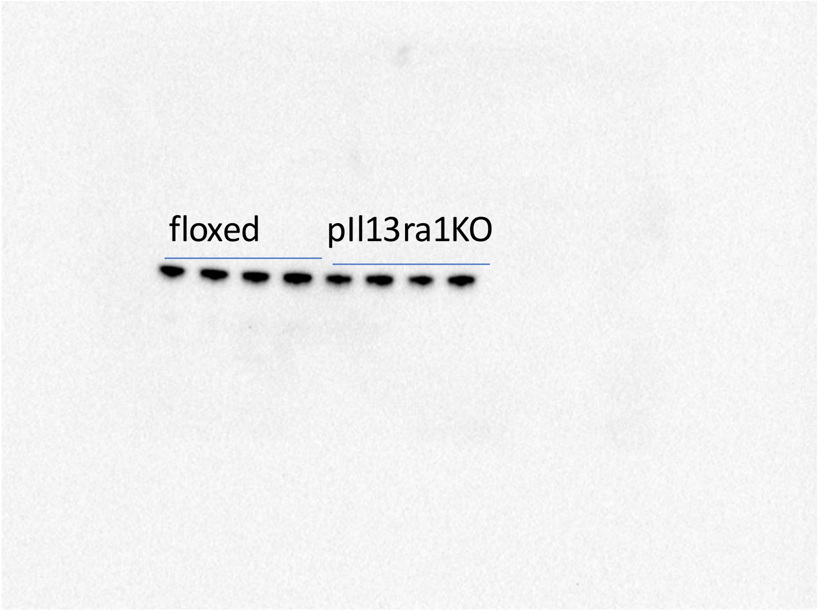

UCP-1

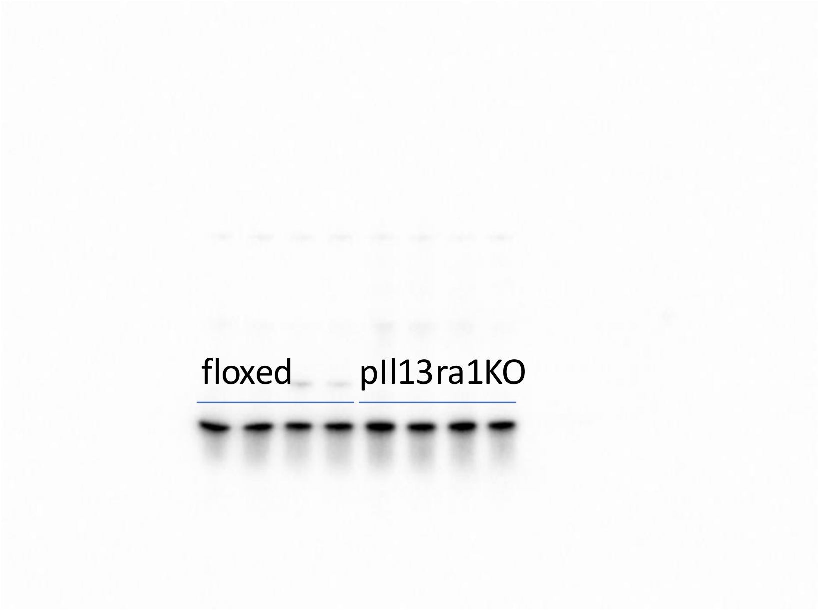

Supplemental Figure 2D

OXPHOS short exposure

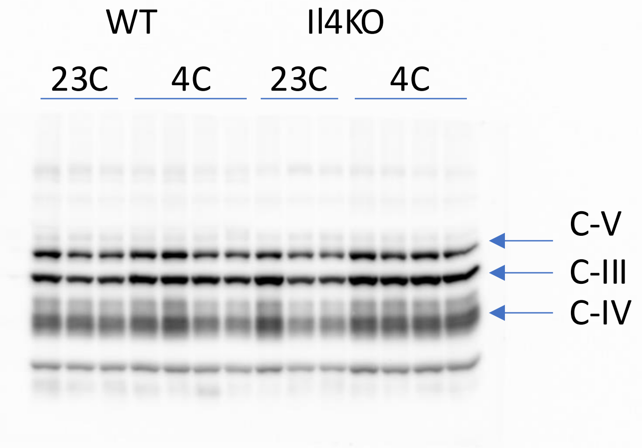

UCP1

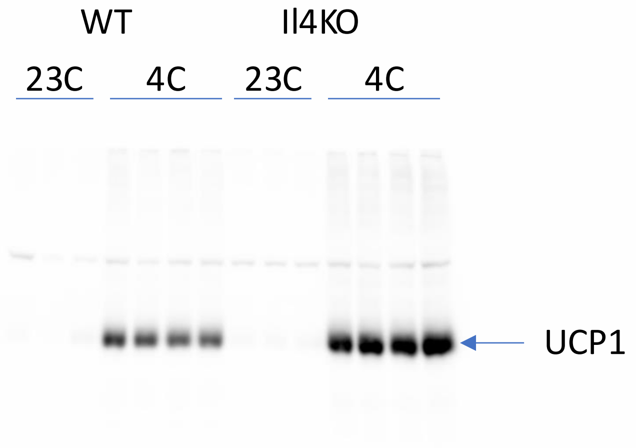

OXPHOS long exposure

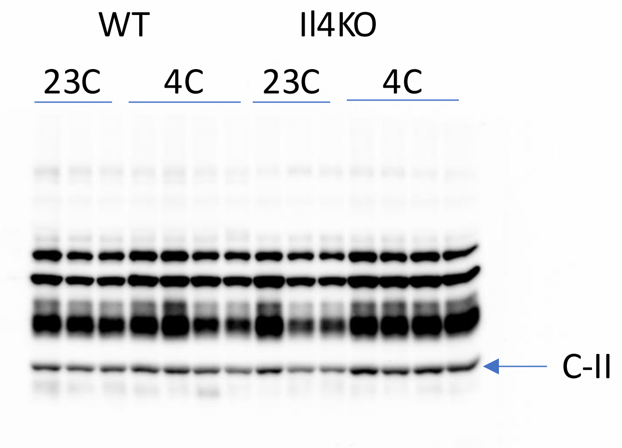

Tubb

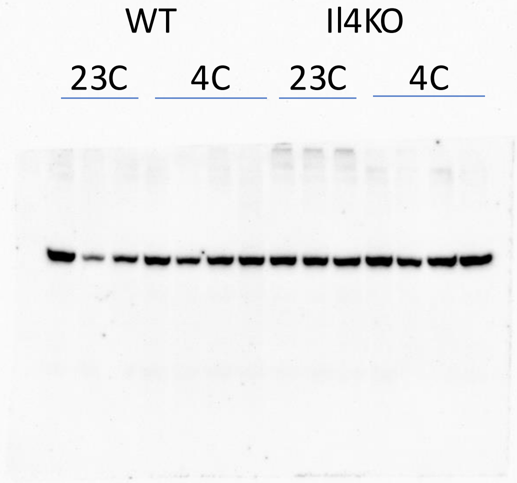

Supplemental Figure 2I

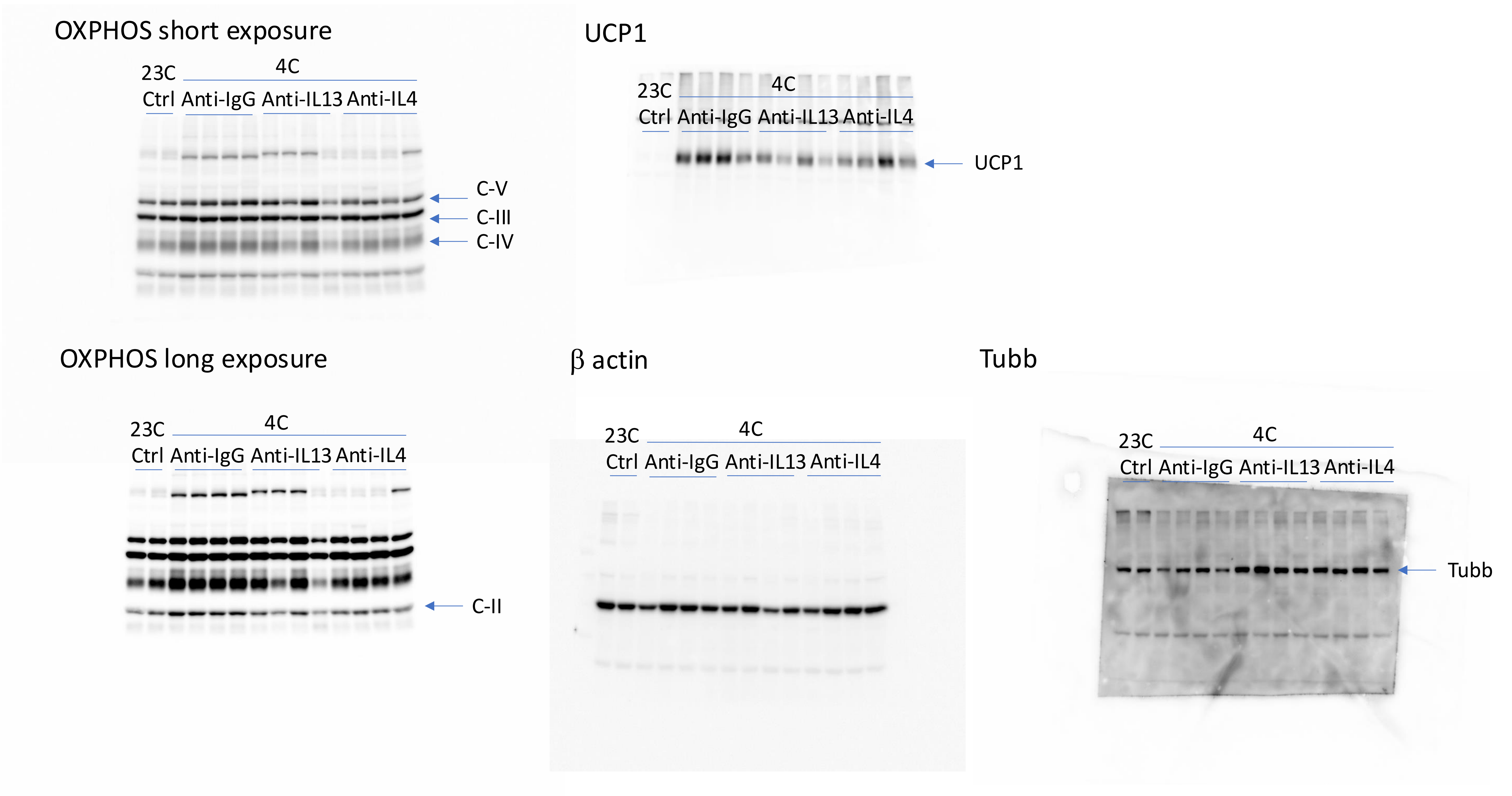

Figure 2C

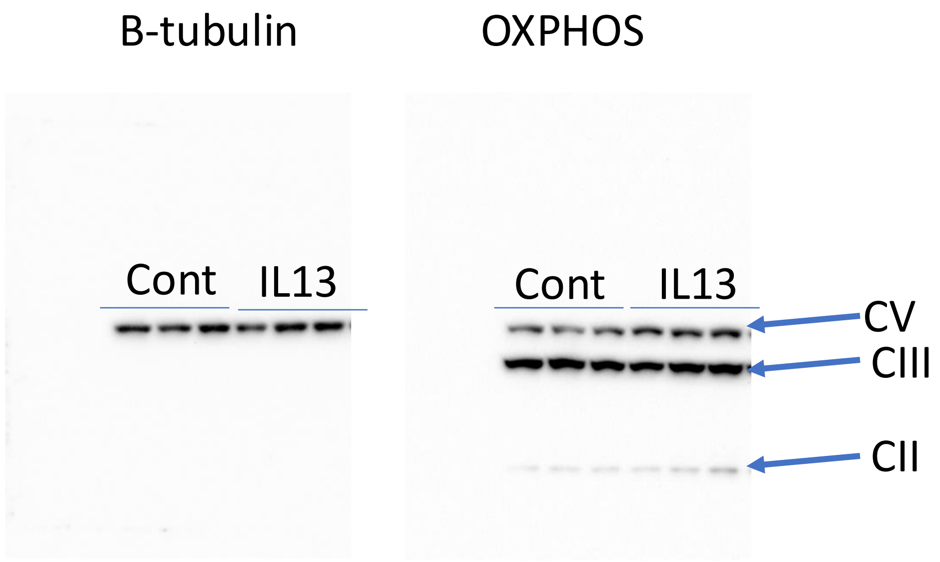

Figure 2D

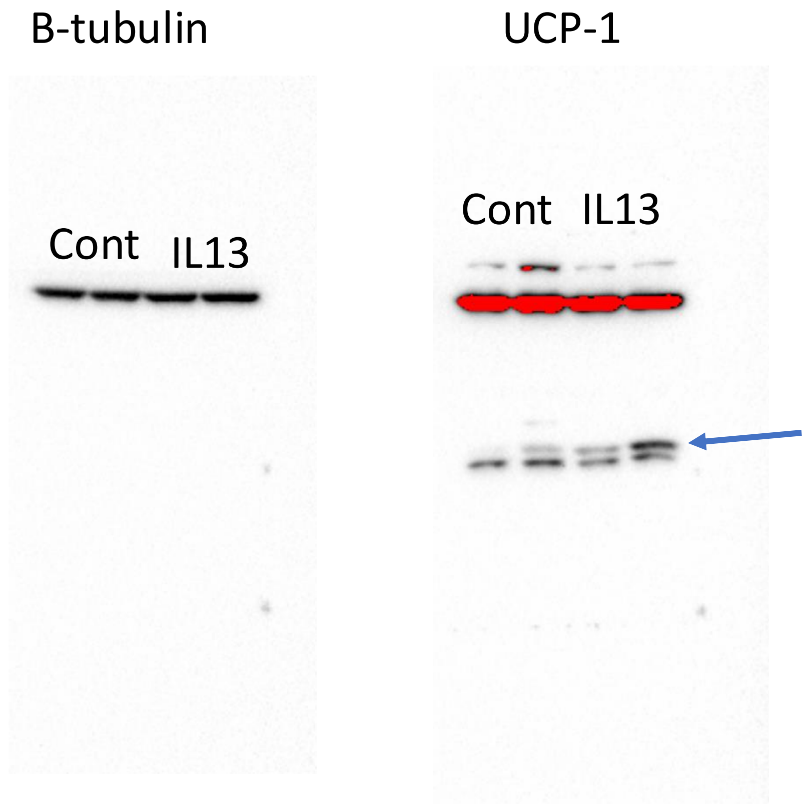

Figure 2H

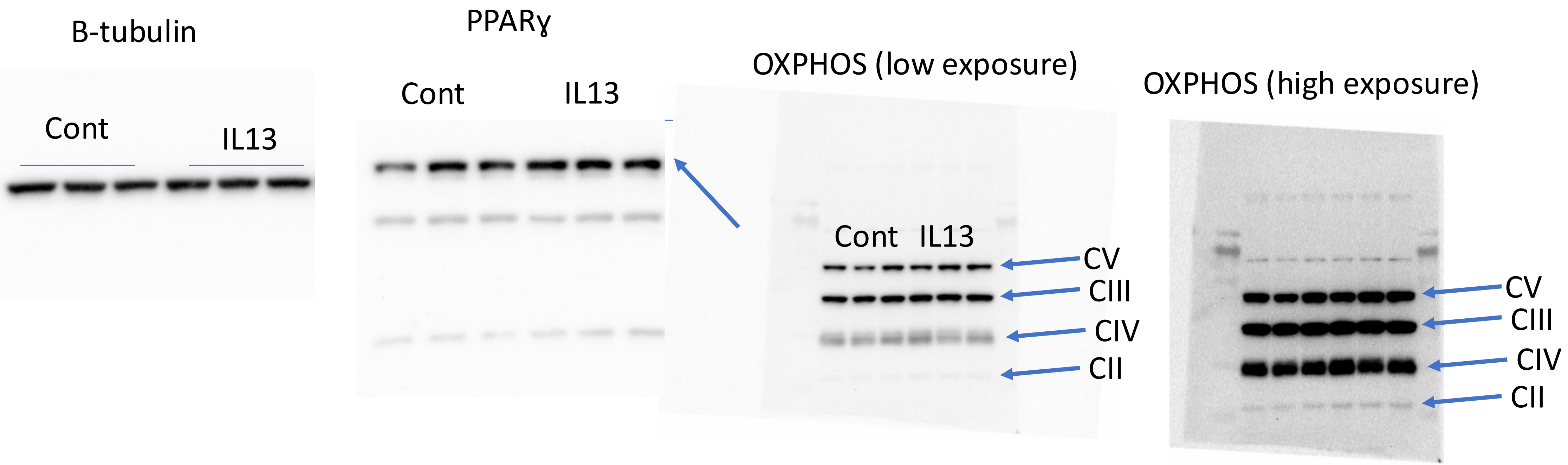

B-tubulin
Cont
IL13
PPARɣ
Cont
IL13
OXPHOS (low exposure)
Cont
IL13
CV
CIII
CIV
CII
OXPHOS (high exposure)
CV
CIII
CIV
CII

Supplemental Figure 3I

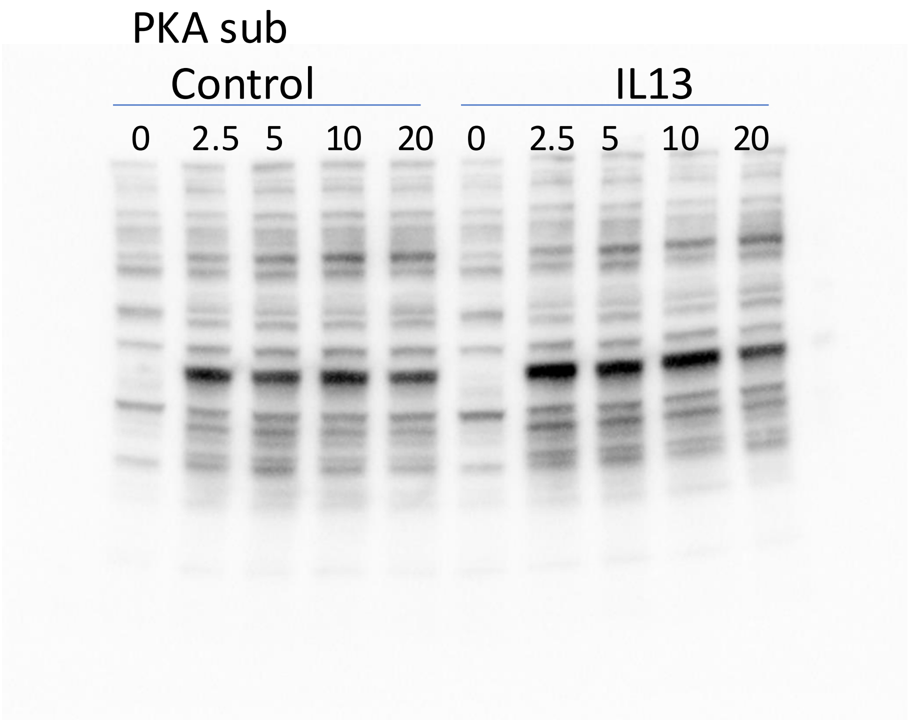

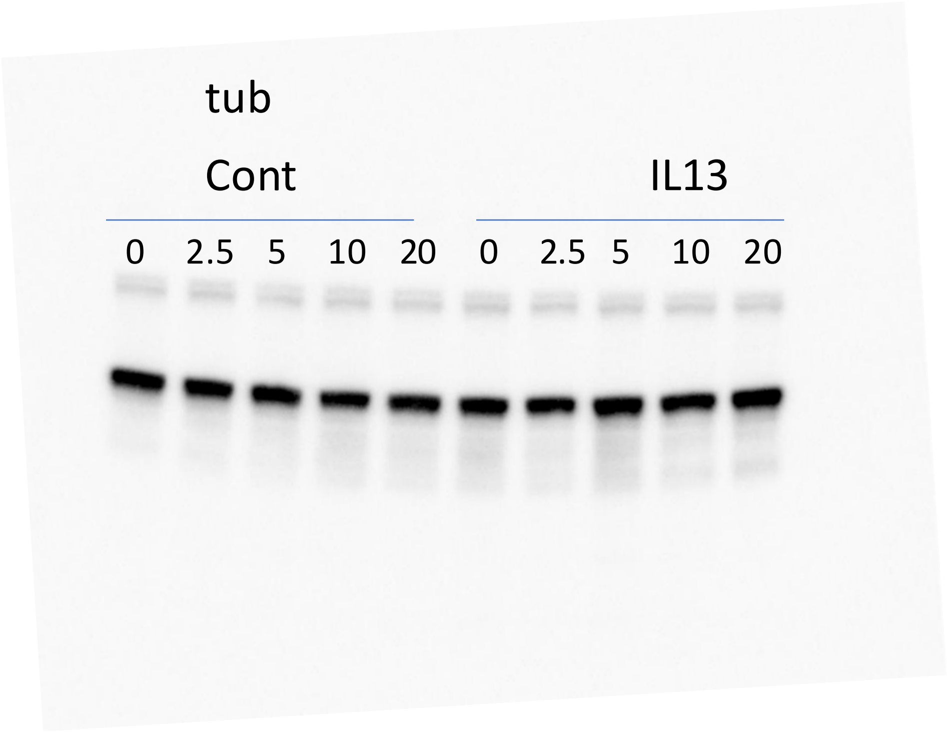

Supplemental Figure 3K

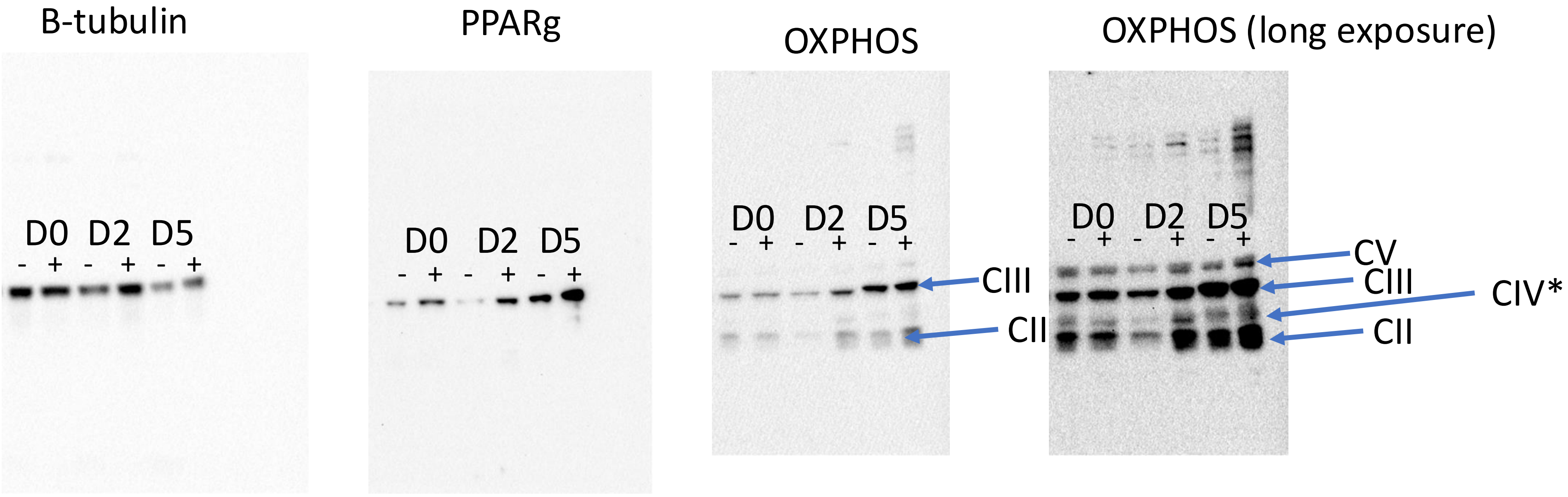

Figure 3A

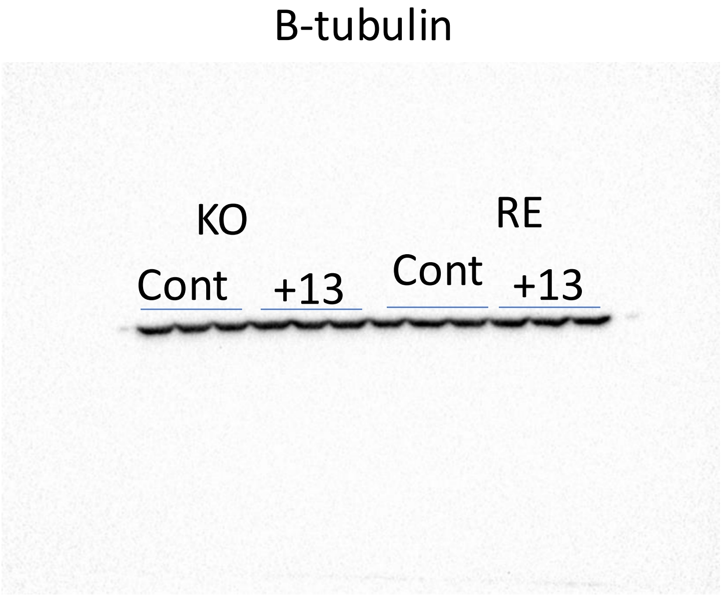

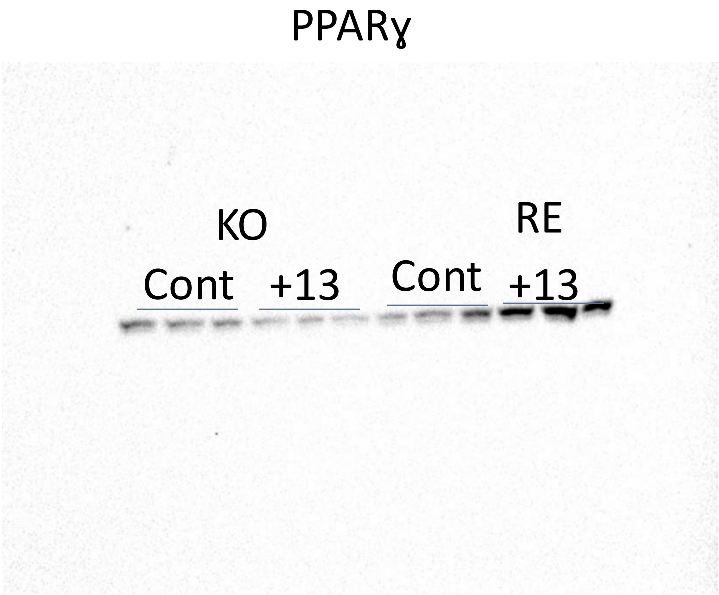

Figure 3E

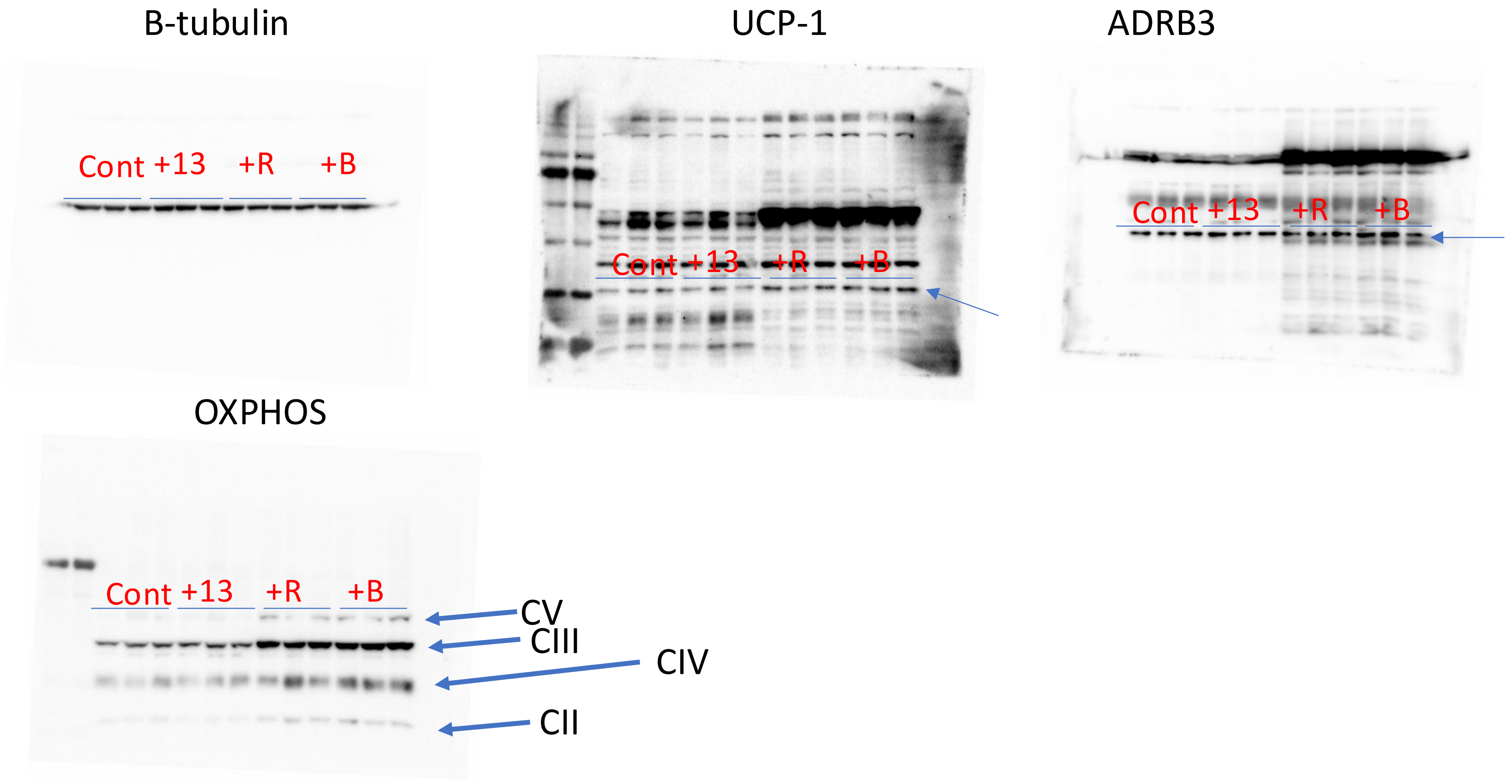

B-tubulin
Cont +13 +R +B
UCP-1
Cont +13 +R +B
ADRB3
Cont +13 +R +B
OXPHOS
Cont +13 +R +B
CV
CIII
CIV
CII

Supplemental Figure 4A

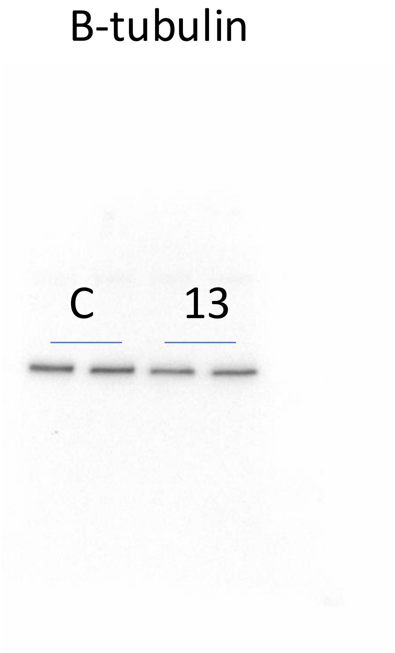

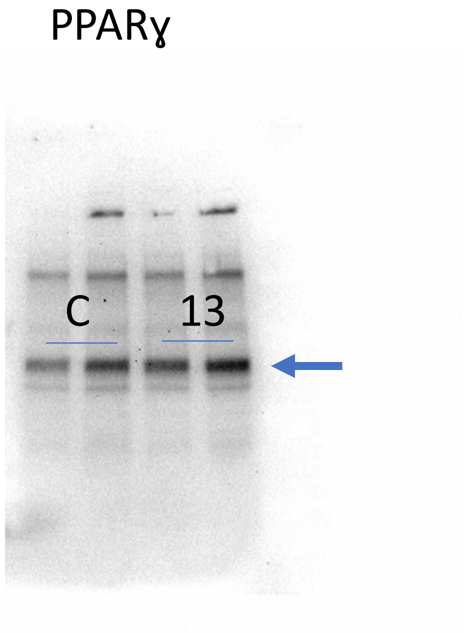

Figure 4G

HSL

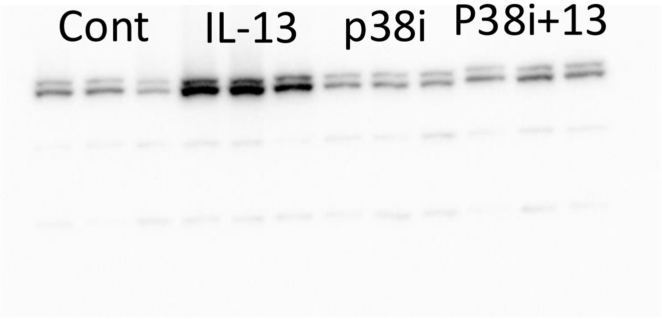

B-tubulin

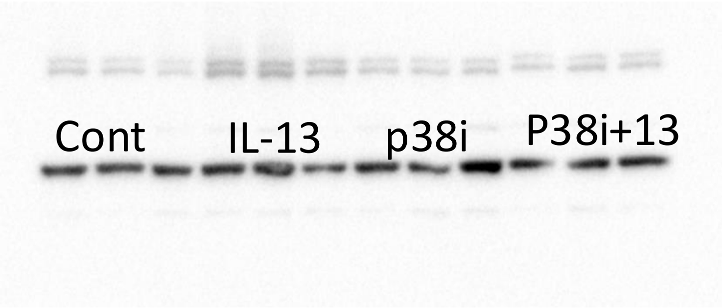

Figure 4H

HSL

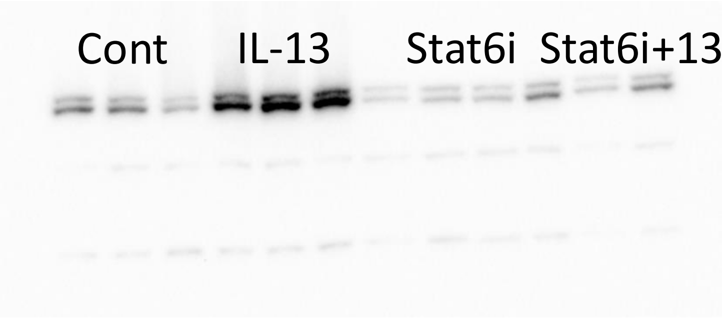

B-tubulin

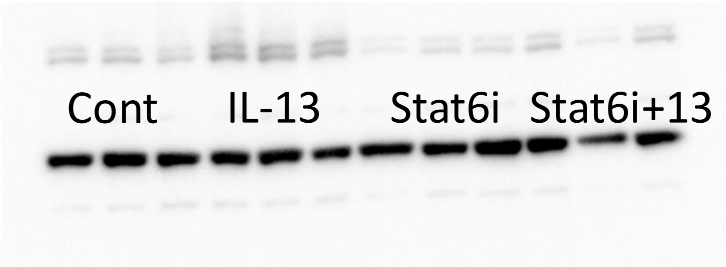

Supplemental Figure 5A

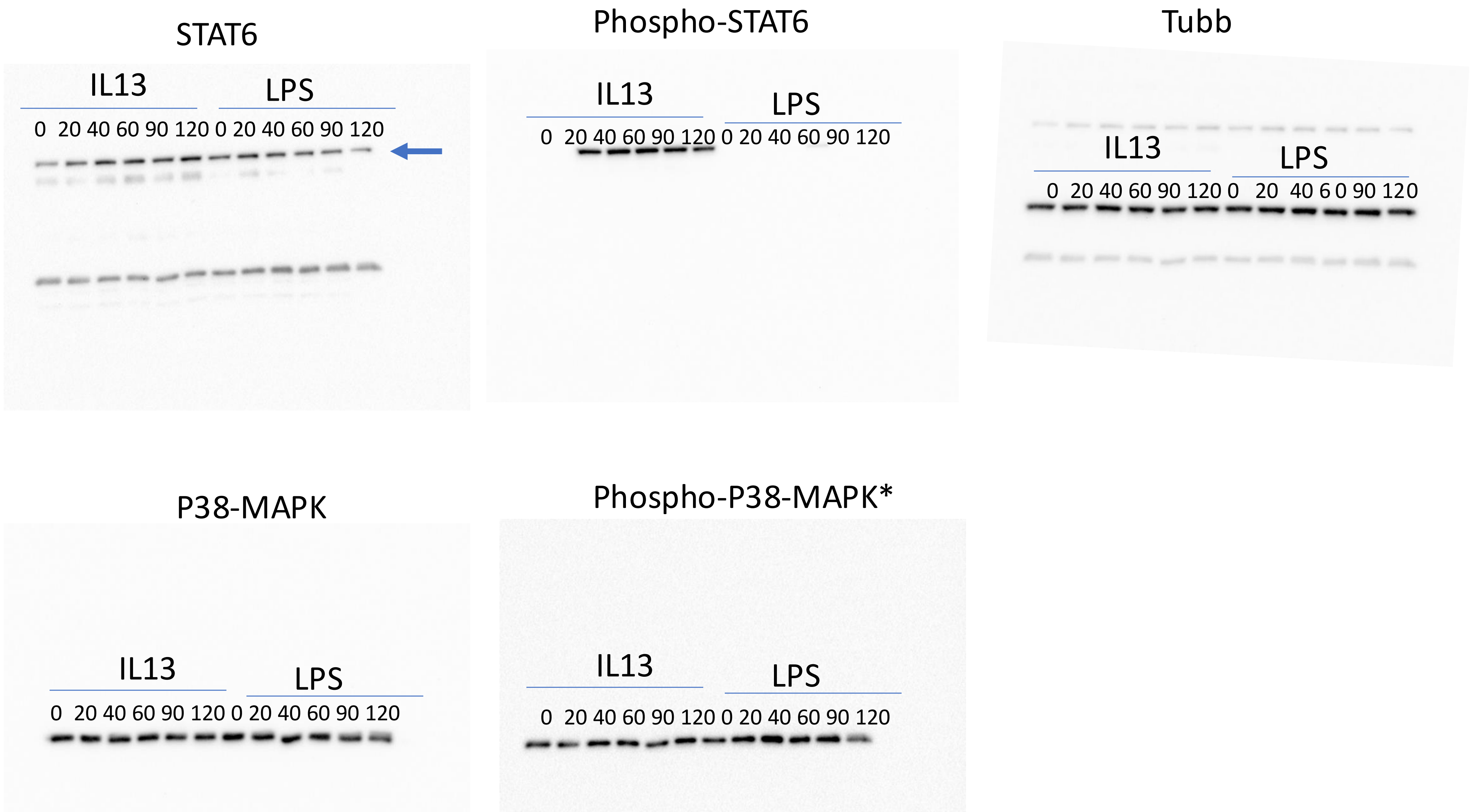

*The entire image in Fig. S4A was brightened up slightly to match the background of other images

Supplemental Figure 5G

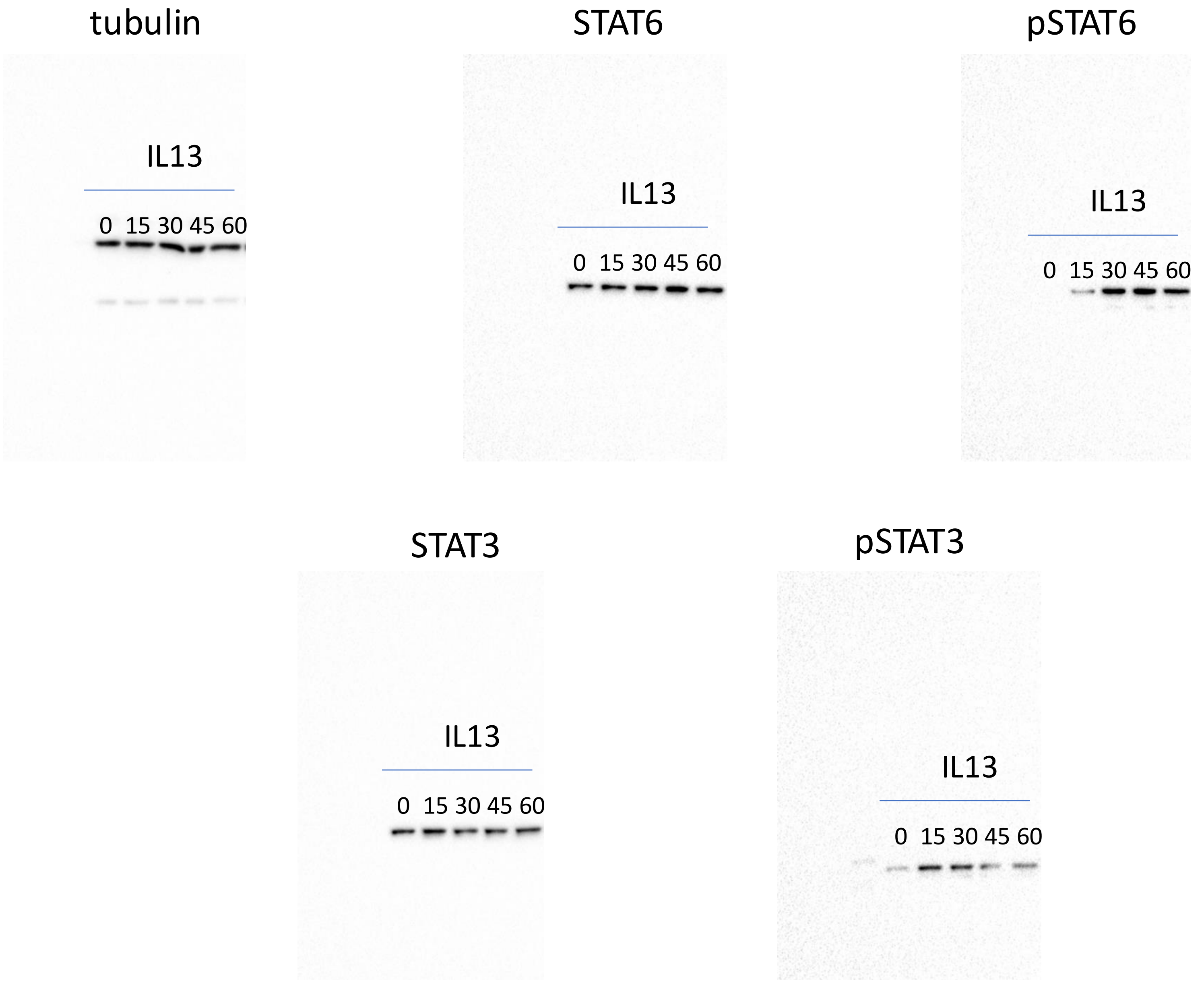

Supplemental Figure 5I

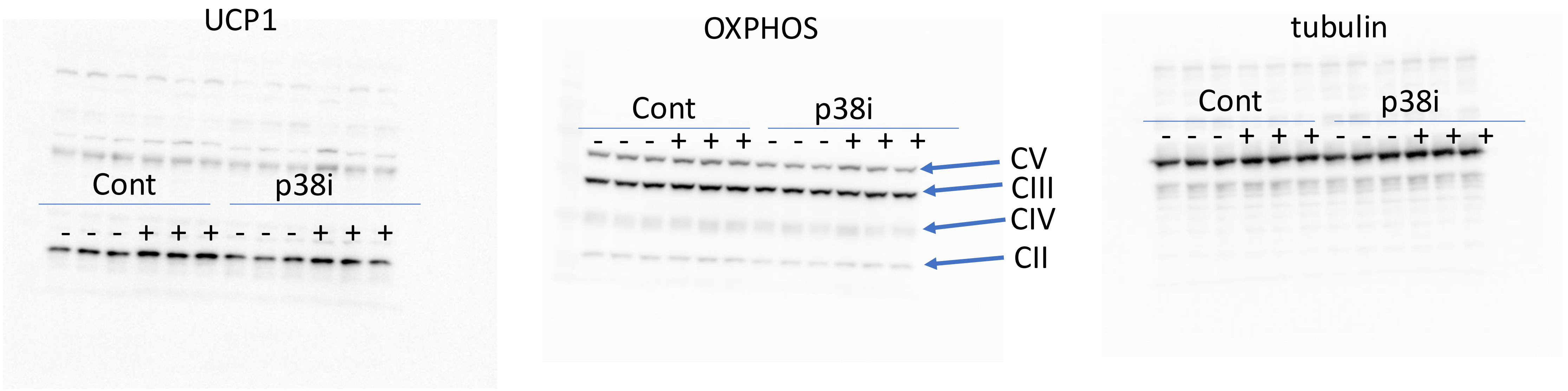

Figure 5B

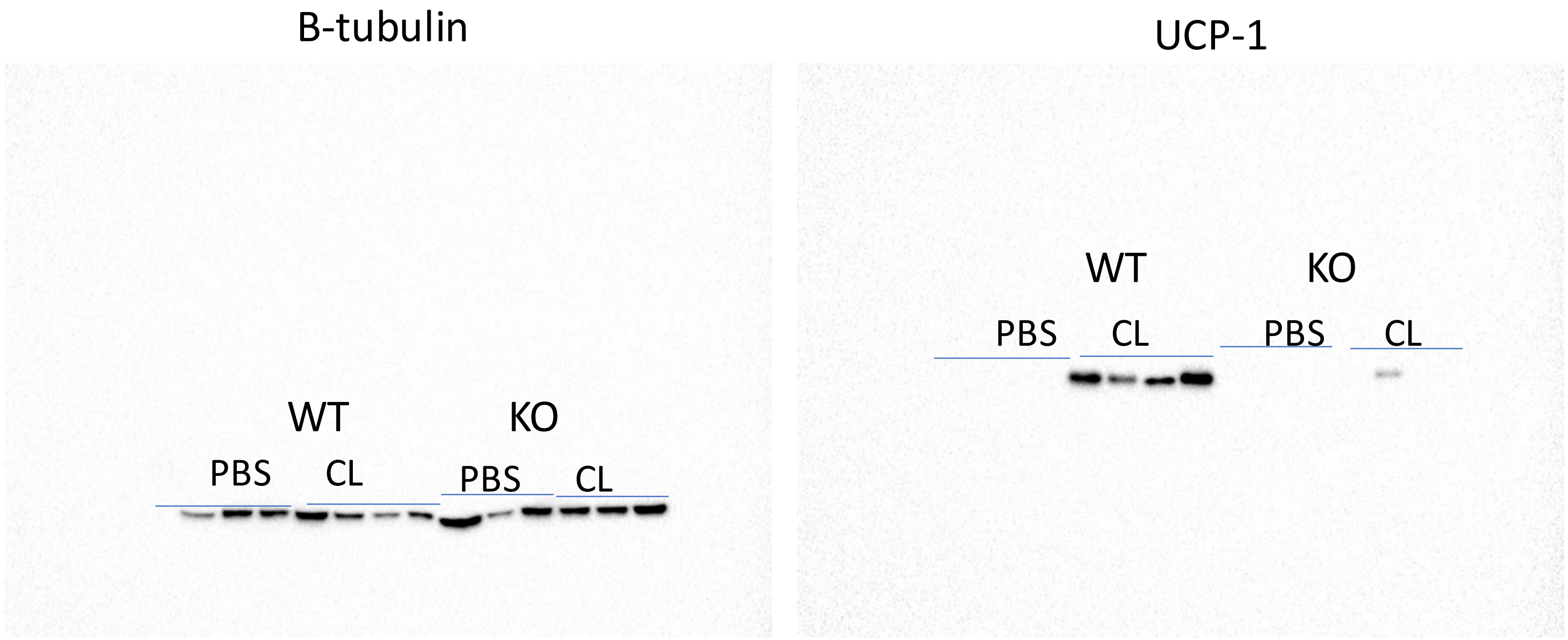

Figure 5D

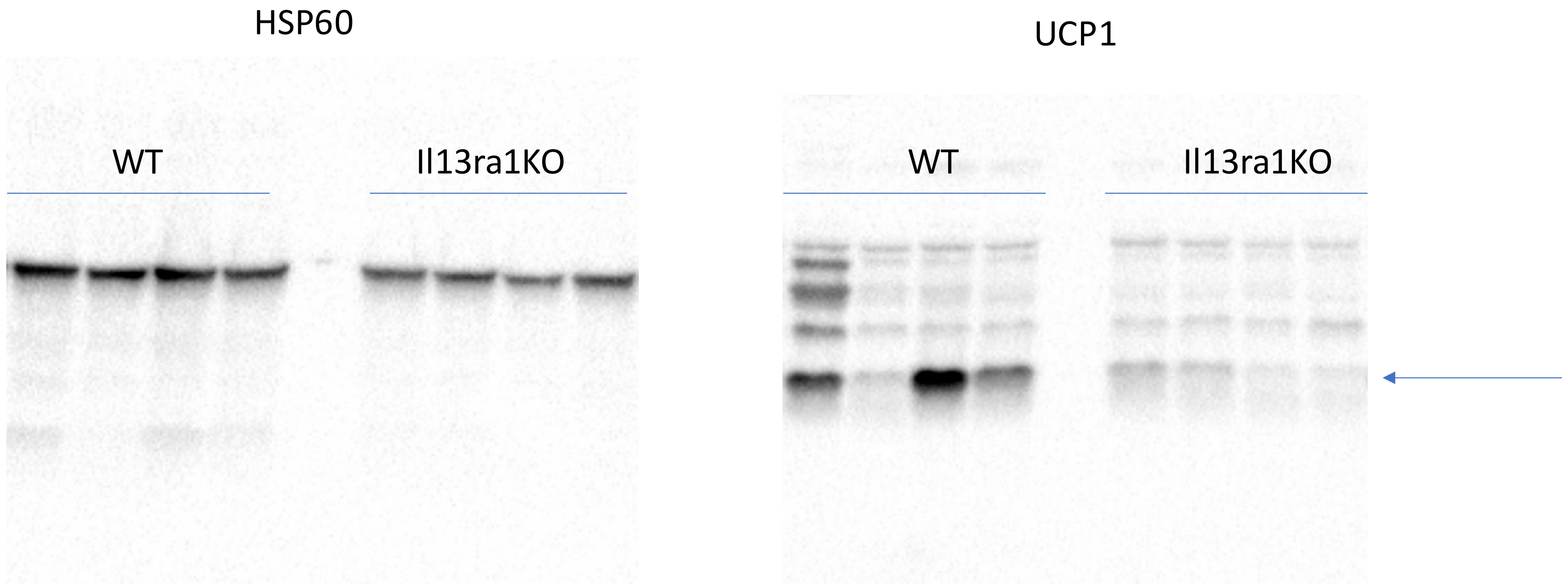

Figure 5F

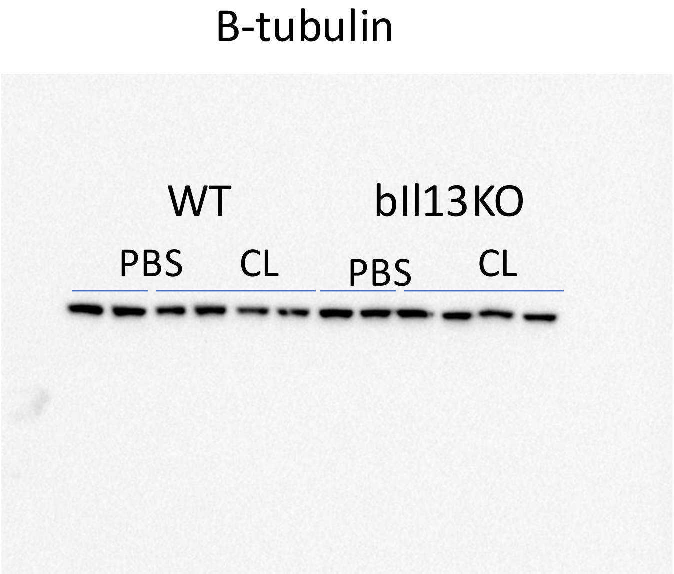
B-tubulin
WT
bIl13KO
PBS
CL
PBS
CL

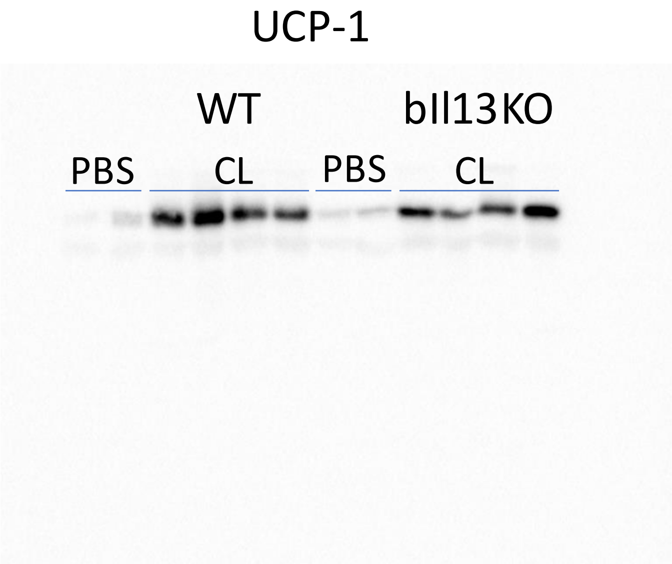
UCP-1
WT
bIl13KO
PBS
CL
PBS
CL

Figure 5G

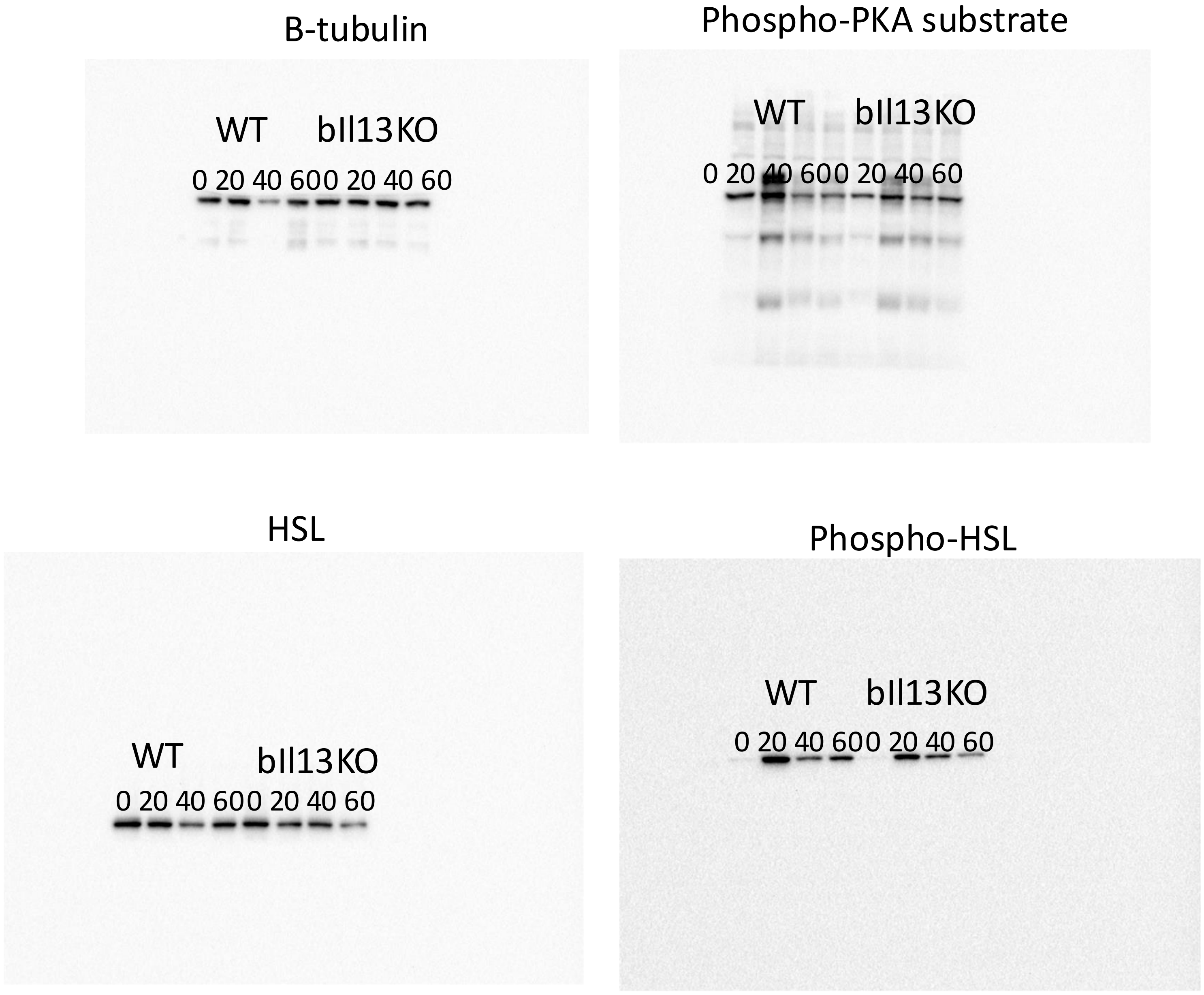
B-tubulin
WT
bIl13KO
0 20 40 600 20 40 60
Phospho-PKA substrate
WT
bIl13KO
0 20 40 600 20 40 60
HSL
WT
bIl13KO
0 20 40 600 20 40 60
Phospho-HSL
WT
bIl13KO
0 20 40 600 20 40 60

Supplemental Figure 6D

B-tubulin

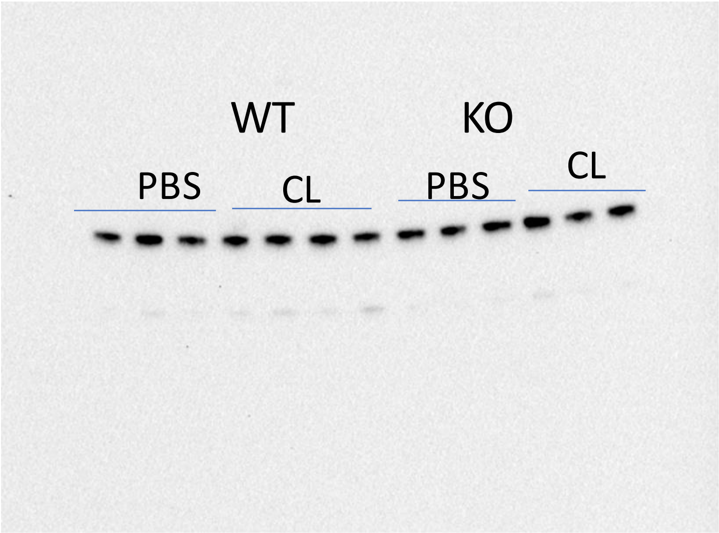

UCP-1

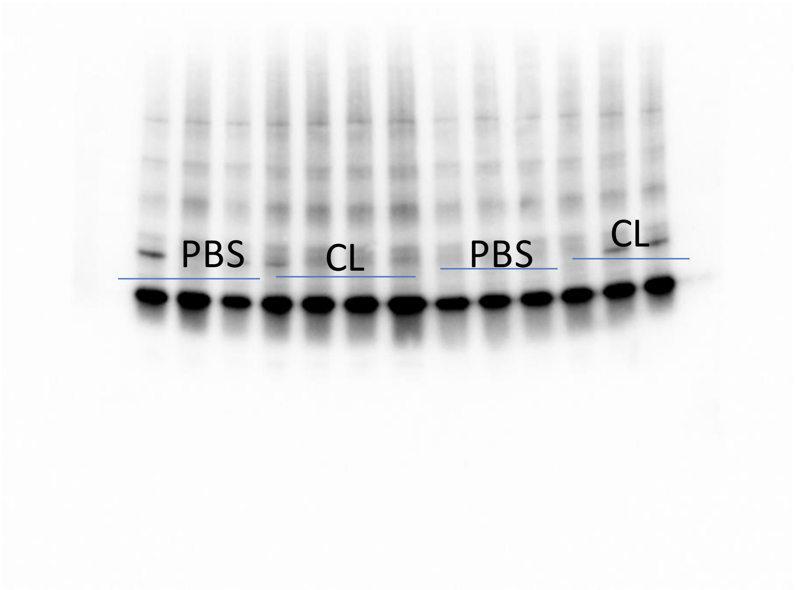

Supplemental Figure 6E

long exposure

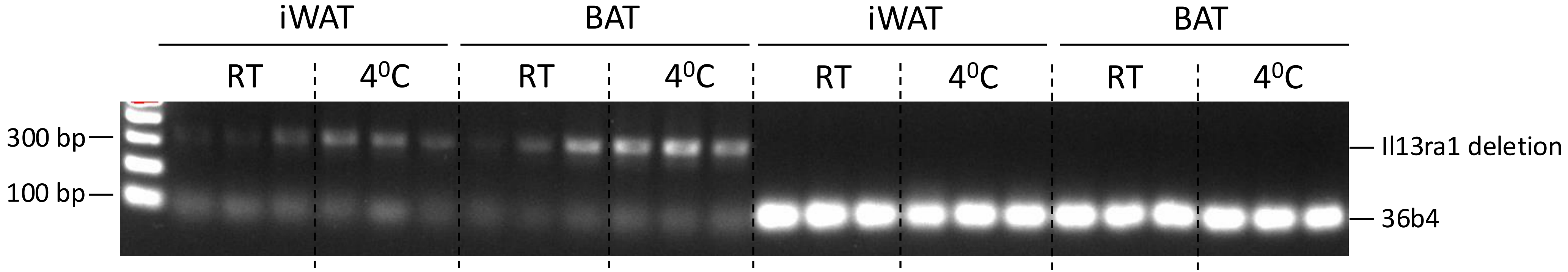

Short exposure

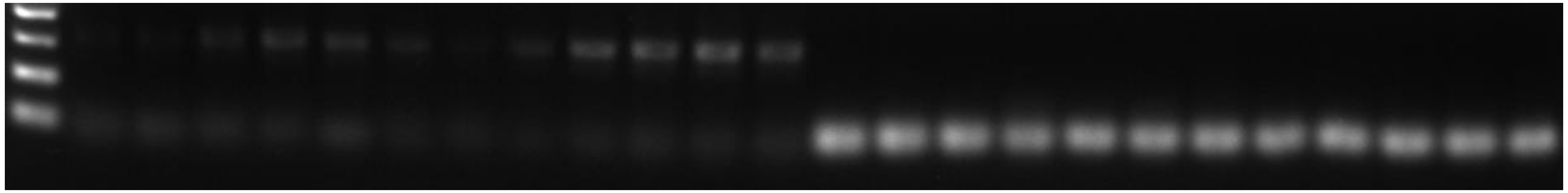

Supplement: Unedited blot and gel images [file jci-135-169152-s157.pdf]
